# A Revised Model for Muscarine Biosynthesis Involving Lysine Trimethylation

**DOI:** 10.1002/anie.7581705

**Published:** 2026-04-23

**Authors:** Sebastian Dörner, Kai Rogge, Felix Trottmann, Christian Hertweck, Dirk Hoffmeister

**Affiliations:** ^1^ Pharmaceutical Microbiology Friedrich Schiller University Jena Jena Germany; ^2^ Leibniz Institute for Natural Product Research and Infection Biology (Hans Knöll Institute) Jena Germany; ^3^ Department Biomolecular Chemistry Leibniz Institute for Natural Product Research and Infection Biology (Hans Knöll Institute) Jena Germany; ^4^ Institute of Microbiology Friedrich Schiller University Jena Jena Germany

**Keywords:** alkaloid, biosynthesis, methyltransfer, muscarine, toxin

## Abstract

l‐(+)‐Muscarine is a widespread fatal toxin produced by various mushrooms that pose a severe threat to human health when they are mistaken for edible species. Apart from a single 1970s study that assumed l‐glutamate and pyruvate were the building blocks of this unusual quaternary amine, surprisingly little is known about the toxin's biogenesis. We used *Collybia rivulosa* (syn*. Clitocybe rivulosa*), a mushroom notorious for producing muscarine, as our model for stable isotope incorporation experiments and subsequent extensive mass spectrometric analysis. Our results provide unambiguous evidence that the backbone of muscarine is assembled from two amino acids, l‐lysine and l‐alanine. Furthermore, we found that iterative ε‐methylation of non‐protein‐bound l‐lysine is the biosynthetic gateway step that yields ε‐*N*,*N*,*N*‐trimethyl‐l‐lysine. This methylation is specific to fungi that produce muscarine. Despite a substrate overlap with the biosynthesis of l‐carnitine, we demonstrate that these two pathways are distinct. Our results provide compelling insight into the biogenetic origin of muscarine and fundamentally revise the previous biosynthetic model for this infamous toxin. The revised biosynthesis model lays the foundation to discover as yet unknown muscarine‐like metabolites that are potentially toxic as well or pharmacologically relevant.

## Graphical abstract

The fatal mushroom toxin l‐(+)‐muscarine is one of the most prominent natural products. We elucidated its biosynthetic origin in the sweating mushroom *Collybia rivulosa* based on incorporation of stable isotope‐labeled compounds and extensive mass spectrometric analyses. We identified l‐lysine and l‐alanine/pyruvate as primary building blocks, thereby fundamentally revising the established biosynthetic model.

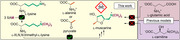

## 1 Introduction

**FIGURE 1:**
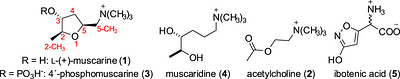
Chemical structures of the neuroactive mushroom natural products l‐(+)‐muscarine (**1**), 4´‐phosphomuscarine (**3**), *rel*‐muscaridine (**4**), of the neurotransmitter acetylcholine (**2**) and the mushroom toxin ibotenic acid (**5**), which occurs in racemic form in the mushrooms. The numbering of atoms in **1** (red) follows Nitta et al. [18].

The infamous mushroom metabolite l‐(+)‐muscarine (**1**, Figure 1) belongs to the most toxic natural products [1, 2]. Its quaternary amine structure mimics the neurotransmitter acetylcholine (**2**, Figure 1), i.e., the intrinsic ligand of muscarinic acetylcholine receptors (mAChRs). Thus, **1** agonistically interferes with the peripheral cholinergic nervous system and causes sweating, dyspnea, bradycardia and circulatory failure. Fatal poisonings occurred—and tragically still do—when **1**‐producing mushrooms, primarily *Clitocybe* species (funnel caps) and species of the genus *Inocybe* (fiber caps), were misidentified as edible species and ingested [1, 3, 4]. Although being a notorious toxin, the janus‐faced character of **1** becomes evident from its positive role in modern pharmacology: the parasympathomimetic properties of **1** were critical to discover chemical cell‐to‐cell signaling, which led to the discovery of the aforementioned receptors, named after **1** [5, 6]. These receptors represent a subfamily of the G protein‐coupled receptors (GPCRs) and are present in the central nervous system, heart, lung, and other organs, therefore regulating numerous physiological functions. The molecular characterization of GPCRs was pioneered with mAChRs [7], and insight into their function has led to the development of various pharmaceuticals [5, 8, 9, 10]. Current research focuses on mAChR antagonists for treating Alzheimer's disease, schizophrenia, diabetes, drug addiction, and cancer [6].

**1** was first isolated from the fly agaric *Amanita* (*A*.) *muscaria*, reported in 1869 [11], yet nearly another century passed before its chemical structure was elucidated in 1957 [12]. To this day, **1** has intrigued generations of organic chemists who have used it as a model compound to develop new methodologies for organic synthesis. Its relevance to chemistry, toxicology, and pharmacology is reflected in the approximately 30 synthetic routes that have been devised to synthesize **1** and its stereoisomers [13, 14]. In contrast, the biological synthesis of **1** has remained mysterious. This is particularly surprising given the toxin's importance and its widespread occurrence, alongside its congeners, 4´‐phosphomuscarine (**3**) and muscaridine (**4**, Figure 1) [15], in the genera *Clitocybe* and *Inocybe*, and others [16, 17].

**SCHEME 1:**
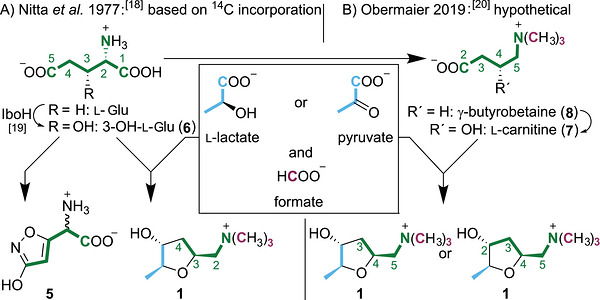
Previous tentative models of l‐(+)‐muscarine (**1**) biosynthesis [18, 20]. The colors illustrate the origin of the respective carbon and nitrogen atoms (dark green: l‐Glu; light blue: l‐lactate/pyruvate; ruby red: formate. Atom numbers in **1**, **7**, and **8** reflect the carbon atoms of l‐Glu. Please refer to Scheme S1 for background information on the metabolic relationship of l‐Glu with **7** and **8**. The dioxygenase IboH is involved in ibotenic acid biosynthesis in the Fly Agaric *Amanita muscaria*.

Only a single publication provides experimental data on a ^14^C radiotracer incorporation study with *Collybia* (*C*.) *rivulosa* (syn. *Clitocybe rivulosa*, the sweating mushroom), conducted by Eugster and his co‐workers [18]. The authors concluded that carbons C2‐C4 and the nitrogen atom of l‐glutamic acid are incorporated into **1** (Scheme 1A), whereas the carboxyl groups at C‐1 and C‐5 are not. Furthermore, they considered all three pyruvate carbon atoms to be the source of the remaining carbon atoms in the backbone of **1**. Supplementation with radiolabled formic acid led to labeled N‐methyl groups which was interpreted as an origin from the cellular single‐carbon pool.

Over four decades later, another biosynthetic model was conceived, based on a study that actually examined the biosynthesis of ibotenic acid (**5**), the major natural product of *A*. *muscaria*. Obermaier and Müller suggested that *threo*‐3‐hydroxy‐l‐glutamic acid (3‐OH‐l‐Glu, **6**), resulting from the stereoselective hydroxylation of l‐Glu, may represent a common precursor of both neuroactive compounds produced by *A. muscaria* (Figure 1 and Scheme 1A) [19]. This biotransformation to **6** is catalyzed by the α‐ketoglutarate‐dependent dioxygenase IboH, but genes encoding IboH‐type enzymes were not found in other **1**‐producing mushrooms. Thus, an alternative hypothesis was put forth assuming the conversion of l‐Glu to l‐carnitine (**7**) as the precursor of **1** (Scheme 1B) [20]. However, this model conflicts with Eugster's incorporation experiments [18], as C‐5 of l‐Glu, which was reported not to be incorporated, would appear as 5‐CH_2_ in **1**, according to the proposed pathways (Scheme S1). Consequently, the biosynthetic origin of **1** has remained enigmatic even though insight into the true biosynthetic sequence may lead to as yet unknown **1**‐like metabolites that may prove highly toxic and/or possess pharmacological relevance.

Here, we present a revised biosynthetic model for the biosynthesis of **1** that is well supported by experimental data, collected from extensive mass spectrometric analyses, synthetic reference compounds, and biotransformation, dismissing both l‐Glu and **7** as precursors. We show an infamous mushroom toxin, which still causes poisonings and, thus, threatens human health, originates from l‐lysine. Furthermore, we report that the iterative methylation of l‐lysine is the gateway biosynthetic event and demonstrate that the **1** and **7** pathways diverge following this initial step.

## 2 Results and Discussion

### 2.1 l‐Glutamic Acid and l‐Carnitine are not Building Blocks of Muscarine

**FIGURE 2:**
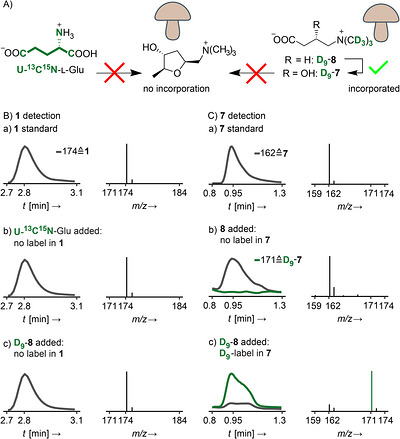
Test of previous biosynthetic models. (A) schematic representation of the tested precursors. Panels B and C show extracted ion chromatograms (EIC) and mass spectra (non‐isotopically labeled **1**: *m*/*z* 174, **7**: *m*/*z* 162). Panel B: chromatograms and spectra to detect **1**. (a) authentic **1**; (b) mycelial extract of a U‐^13^C^15^N‐l‐Glu‐supplemented culture (to test the model by Nitta et al.; (c) mycelial extract of a D_9_‐**8**‐supplemented culture (Obermaier's model). Panel C: chromatograms and spectra to detect **7**; (a) authentic **7**; (b) **7** detected in a control extract of a culture supplemented with unlabeled **8**; (c) deuterated **7** (*m*/*z* 171), detected in an extract of a culture supplemented with D_9_‐**8**.

Building on Eugster's pioneering work [18], we sought to narrow down the possibilities how the biosynthetic sequence proceeds and to identify further compounds associated with the assembly of **1**. Initially, our goal was to test whether l‐Glu is incorporated into **1** and serves as the main origin of its backbone. To this end, we supplied U‐^13^C^15^N‐l‐Glu to **1**‐producing cultures of *C. rivulosa*. This species, which was also used by Eugster in the prior study [18], can be grown as mycelial cultures in a laboratory setting under reproducible conditions. We monitored the culture extract by UHPLC‐MS and used an authentic standard of unlabeled **1** as reference (Figure 2).

We chromatographically identified **1** in the methanolic fungal extract (Figure 2B). Unexpectedly, mass spectrometry did not indicate any incorporation of a stable isotope from l‐Glu, which is inconsistent with the published biosynthetic scheme (Scheme 1) [18]. Next, we tested Obermaier's hypothesis of **7** incorporation. Due to its zwitterionic character, the capacity of *C. rivulosa* to take up **7** was unclear. Therefore, we added γ‐butyrobetaine (**8**) as key precursor, rather than **7**, to the mycelial cultures to confirm its incorporation into **7** and possibly into **1**. We synthesized methyl‐D_9_‐**8** and tested whether the deuterium label would appear in fungal metabolites. UHPLC‐MS analysis of the mycelial extracts did not indicate any incorporation into **1** (Figure 2B). However, compared to a control with unlabeled **8**, the mass of **7** (eluting at 0.95 min, chromatogram of authentic standard shown in Figure 2C) had increased by nine mass units (*m/z* 171, 162 + 9 *m_u_*) upon addition of D_9_‐**8** to the fungal culture (Figure 2C). This finding indicated that **8** had been taken up by the cells and metabolized to **7** which, however, did not serve as a precursor of **1**. Therefore, neither of the published models was supported, also keeping in mind, that the precision of methods and instruments used to develop Eugster's model may not have met today's standards.

### 2.2 Identification of l‐Lysine as the Major Building Block of Muscarine

**FIGURE 3:**
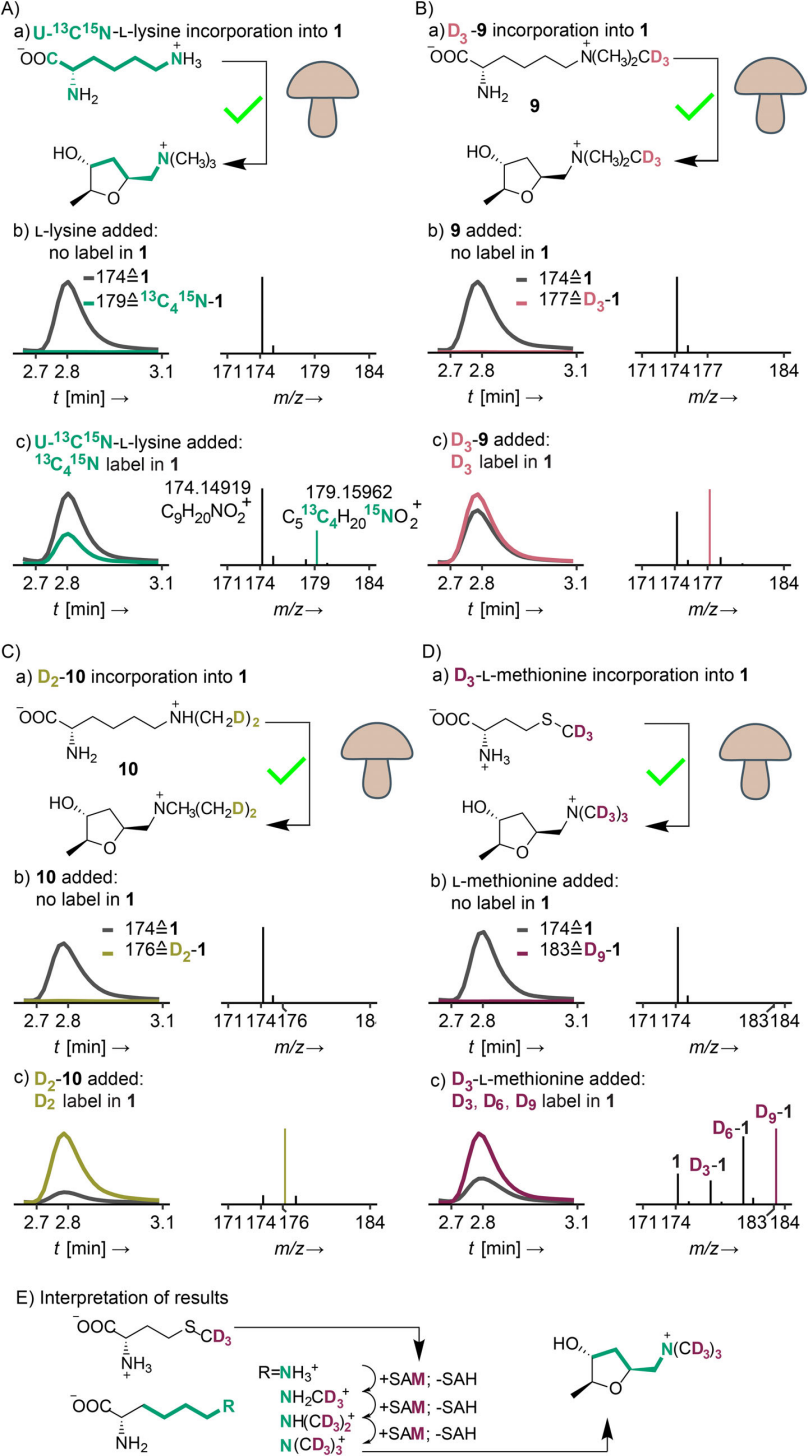
Mass spectrometric evidence for l‐lysine as building block and for initial methyl transfer during **1** biosynthesis. Shown are EICs (left) and mass spectra (right) of extracted *Collybia rivulosa* mycelia, cultured in the presence of the following unlabeled or stable isotope‐labeled compounds. Panel (A) l‐Lys/U‐^13^C^15^N‐l‐Lys; panel (B) **9**/D_3_‐**9**; panel (C) **10**/D_2_‐**10**; panel (D) l‐Met/D_3_‐l‐Met. The reactions leading to stable‐isotope incorporation into **1** are shown in the respective insets designated with (a). Panel (E) displays the summary of the l‐lysine‐ and SAM‐derived portions and their final position in **1**.

In the biosynthesis of **7**, l‐lysine is trimethylated and supplies a C_4_‐N(CH_3_)_3_ moiety which represents a partial structure of **1** as well. Following a more exploratory approach along Obermaier's hypothesis, we further investigated this pathway whose final products, **8** and **7**, had now been shown not to participate in the biosynthesis of **1**. Yet, an earlier intermediate could hypothetically be routed towards **1** assembly, which in return implies l‐lysine as origin for **1**. For stable isotope labeling, U‐^13^C^15^N‐l‐lysine was added to cultures of *C. rivulosa*. Chromatographic and mass spectrometric analysis of culture extracts revealed that the *m*/*z*‐value of **1** had increased by 5 *m_u_* (Figure 3A). A subsequent HR‐ESIMS analysis traced this increase back to four carbon atoms and one nitrogen atom: *m*/*z* 174.14919 [*M* ^+^] (calcd. for C_9_H_20_O_2_N^+^; Δ ‐1.228 ppm, compatible with **1**) and *m*/*z* 179.15962 [*M* ^+^] (calcd. for C_5_^13^C_4_H_20_O_2_^15^N^+^; Δ ‐1.328 ppm, compatible with ^13^C_4_^15^N‐**1**). This result demonstrated the same heavy‐isotope incorporation with **1** as found for the **7** pathway when feeding U‐^13^C^15^N‐l‐lysine.

### 2.3 l‐Lysine Trimethylation Initiates Muscarine Biosynthesis

ε‐*N*,*N*,*N*‐Trimethyl‐l‐lysine (TML, Figure 3B, **9**) is the first intermediate of **7** biosynthesis and emerges from peptidyl‐TML, i.e., a polypeptide from which **9** is liberated to provide the monomer [21]. We synthesized **9** and ε‐*N*‐methyl‐D_3_,ε‐*N*,*N*‐dimethyl‐l‐lysine (D_3_‐**9**) from Boc‐l‐lysine (see Supporting Information) and supplemented fungal cultures with **9** and D_3_‐**9**. Compared to the unlabeled control, an incorporation into **1** was unequivocally shown, as evident by the signal *m*/*z* 177 (174 + 3 *m_u_*, Figure 3B). Considering that this particular origin might limit the control of the metabolic flux and general availability of **9**, we hypothesized that monomeric l‐Lys could function as substrate for the production of **9** to supply the biosynthesis of **1**.

To test this hypothesis, ε‐*N*,*N*‐dimethyl‐l‐lysine (**10**) and methyl‐deuterated ε‐*N*,*N*‐dimethyl‐D_2_‐l‐lysine (D_2_‐**10**) were synthesized and used to amend the medium of *C. rivulosa* cultures. As **10** is not a substrate for cellular protein synthesis, this experiment discriminated whether free or protein‐bound lysine is methylated.

The signal *m*/*z* 176 (174 + 2 *m_u_* Figure 3C) demonstrated that TML synthesis in *C. rivulosa* relies on free l‐lysine. As the reaction is likely catalyzed by an *S*‐adenosyl‐l‐methionine (SAM)‐dependent methyltransferase, l‐Met was the expected origin of the methyl groups for **1** biosynthesis. Using l‐methionine‐(*methyl*‐D_3_), the set of signals at *m/z* 174/177/180/183 proved successive stable isotope incorporation of three deuterium atoms per transfer (174 + 0, + 3, + 6, + 9 *m_u_*, Figure 3D).

A recent study on the evolution of the mushroom family Clitocybaceae (funnel caps) showed **1** producing species are restricted to one particular subgenus (*Collybia*, Figure S1) with the notable exception of the evolutionarily most ancient species in this subgenus, *Collybia odora*, that does not produce **1** [22]. Therefore, it is a key species to test the biotransformation of **10** to **9**. Other species of this family and related producers were investigated, as well as non‐producers (Figure S2). In **1**‐producing species, primarily D_2_‐**9** (*m/z* 191, 189 + 2 *m_u_*) was detected, with a lower abundance of **9** (*m/z* 189), when grown in the presence of D_2_‐**10**. In contrast, D_2_‐**9** was only detected in traces in all **1** non‐producing species. Even though only a few species were investigated, our findings support the notion that iterative ε‐methylation of free l‐lysine represents the biosynthetic gateway step en route to **1**. Although this particular methyltransfer reaction appears specific to **1** producing fungi, amino acid trimethylation as initial event of a more complex pathway is familiar from ergothioneine [23].

### 2.4 Delimitation of the Muscarine and the Carnitine Pathways

**FIGURE 4:**
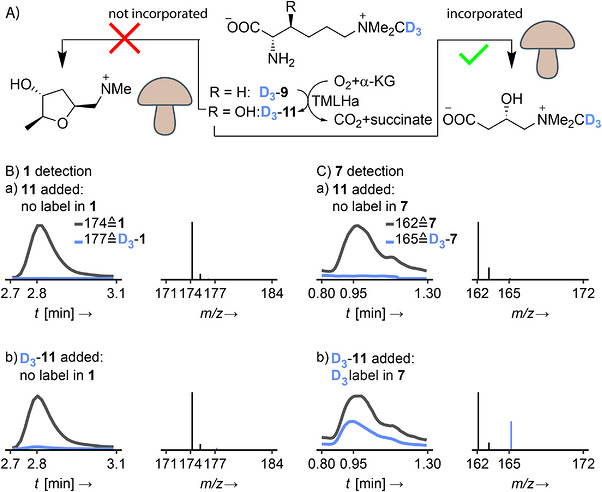
Biosynthetic divergence of the **1** and **7** pathways. Shown are EICs (left) and mass spectra (right) of extracted *C. rivulosa* mycelia, supplied with **11** (for control) or D_3_‐**11**. (A) Enzymatic synthesis of **11** and interpretation of feeding experiment. Acronyms: TMLHa: trimethyl‐l‐lysine hydroxylase, α‐KG: α‐ketoglutarate. (B) Incorporation of **11** or D_3_‐**11**, respectively, into **1**; (C) incorporation of **11** or D_3_‐**11** into **7**.

Both **1** and **7** biosynthesis share **9** as an intermediate, though produced from a monomeric substrate in the former and liberated from proteins in the latter case. We investigated whether the subsequent intermediate of the **7** pathway, 3‐hydroxytrimethyl‐l‐lysine (**11**), is a possible shared intermediate as well. The hydroxylation of **9** at the 3 position allows for further processing of a C_4_N(CH_3_)_3_ fragment by retro‐aldol cleavage of the Cα‐Cβ bond, generating 4‐(trimethylamino)butanal (**12**, Figures 4 and S3). The Incorporation of a C_4_N(CH_3_)_3_ fragment corresponds to the observed incorporation of l‐Lys into **1**. We enzymatically produced **11** and D_3_‐**11** from **9** and D_3_‐**9**, respectively, using human trimethyllysine hydroxylase [24, 25]. The incorporation of **11** into **7** was monitored (Figure 4) to confirm the uptake into the cells. Although D_3_‐**11** did show a clear incorporation into **7**, only a negligible incorporation into **1** was observed, possibly due to trace impurities of D_3_‐**9** (1.8 % AUC of D_3_‐**11**). Hypothetically, the cleavage of the Cα‐Cβ bond of **11** may also proceed through its 3*R* diastereomer. This scenario would allow to differentiate the **1** and **7** pathways in the fungal cell, yet generate **12** (Figure 4) as shared cleavage product. This intermediate of the **7** pathway was also tested by synthesizing and amending the medium with **12** and D_3_‐**12**, respectively. While incorporation into **7** was observed, this was not the case for **1** (Figure S3). These results prove the biosynthetic pathways of **1** and **7** follow separate routes, diverging from **9** as last common intermediate.

### 2.5 l‐Alanine/Pyruvate as Origin of Carbons C2 and 2‐CH_3_

**FIGURE 5:**
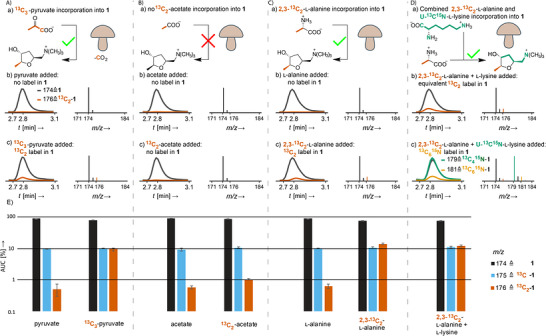
Stable‐isotope labeling to identify the origin of carbon atoms C2 and 2‐CH_3_ in **1**. Panels A‐D depict the found or disproven stable‐isotope incorporation reaction into **1** (top). Below, EICs (left) and mass spectra (right) of extracted *C. rivulosa* mycelia, cultured in the presence of unlabeled compounds for control or stable isotope‐labeled compounds are shown. Added compounds: pyruvate (panel A); acetate (B); l‐alanine (C); l‐alanine + l‐lysine (D). Panel E: Quantification of stable‐isotope incorporation in **1**. Bar diagrams show average percent of the areas under curve of *m*/*z* 174, 175, and 176 peaks. Note the logarithmic y‐axis. Labeled compounds or unlabeled controls were added at 5 mm final concentration to the cultures. Error bars indicate the standard deviation (*n* = 3). For additional data on labeled pyruvate feeding, please see Figure S4.

As l‐lysine contributes only four of the six carbon atoms to the backbone of **1**, we investigated the possible origin of the remaining two carbons, i.e., C2 and 2‐CH_3_. Prior work by Eugster and colleagues [18] traced C2 and 2‐CH_3_, as well as C3, back to pyruvate which corresponds to the entire pyruvate molecule, i.e., to atoms C2, C3, and C1, respectively (Scheme S1). Strikingly, the incorporation of 2‐^14^C‐pyruvic acid showed an incorporation rate 3.8 times higher than that of 1‐^14^C‐pyruvic acid. With our revised **1** biosynthesis model in mind and l‐lysine as identified precursor, we hypothesized that pyruvate carbons C2 and C3 may in fact be supplied to **1** assembly. To test this hypothesis, we quantified the incorporation of U‐^13^C‐pyruvate and 1‐^13^C‐pyruvate, alongside a non‐labeled control, into **1** (Figure 5A). 1‐^13^C‐pyruvate showed negligible incorporation into **1** (Figure S4 and Table S1). However, a moderate, yet clear incorporation was observed for U‐^13^C‐pyruvate, as evident by an increase by two mass units (*m/z* 176, 174 + 2 *m_u_*). As acetate, derived from U‐^13^C‐pyruvate, would also carry the C2‐ and C3‐^13^C stable‐isotope label, we additionally investigated the incorporation of 1,2‐^13^C‐acetate (Figure 5B), yet observed merely a negligible increase by two mass units, compared to the unlabeled acetate experiment. This makes acetate—as a follow‐up product of pyruvate—unlikely as the origin of C2 and 2‐CH_3_ atoms in **1**.

As pyruvate is readily converted into l‐alanine, we further tested the incorporation of 2,3‐^13^C_2_ ‐l‐alanine, together with an unlabeled control (Figure 5C). Compared to U‐^13^C‐pyruvate (9.45 ± 1.07 %), 2,3‐^13^C_2_ ‐l‐alanine (13.92 ± 0.71 %) showed higher incorporation, suggesting pyruvate first required amination to l‐alanine for biosynthesis in **1**.

The above‐described results virtually excluded the possibility that the observed stable‐isotope integration was due to pyruvate incorporation via acetate/acetyl‐CoA into l‐lysine, and thus into positions of **1** other than C2 and 2‐CH_3_. To rule out unspecific incorporation of the pyruvate stable‐isotope label, we supplied 2,3‐^13^C_2_‐l‐alanine in the presence of additional unlabeled and U‐^13^C^15^N‐l‐lysine (Figure 5D) to saturate the cells and, hence, eliminate the need for de novo l‐lysine biosynthesis.

Even in this experimental setup, a comparable incorporation rate of the two l‐alanine‐derived ^13^C atoms was found. This result strongly suggests that the stable‐isotope label introduced into **1** by l‐alanine is independent of that via l‐lysine. Furthermore, a +7 amu peak was detected when both 2,3‐^13^C_2_‐l‐alanine and U‐^13^C^15^N‐l‐lysine were supplied simultaneously, suggesting a fully labeled carbon and nitrogen **1** backbone (Figure 5D). The assignments of the incorporated stable isotope‐labeled atoms to particular positions in **1** were supported by additional MS/MS experiments (Figure S5 and Table S2). Compared to l‐lysine, the lower incorporation rate of l‐alanine is presumably either due to modest uptake of l‐alanine into the cells or due to higher intracellular titers of endogenous unlabeled l‐alanine, derived from pyruvate from the primary metabolism (Figure 5E). In fact, cellular glycolysis produces large amounts of pyruvate that is not subject to end product inhibition, as opposed to l‐lysine biosynthesis [26].

**FIGURE 6:**
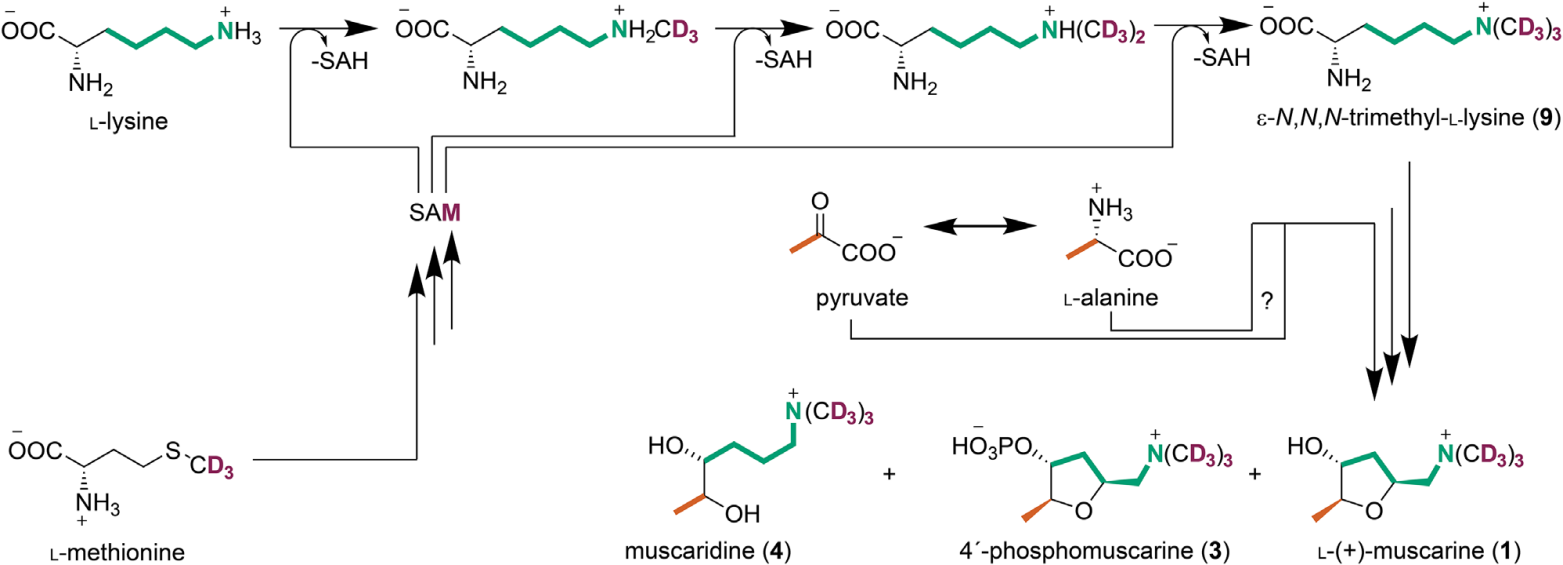
Revised biosynthesis of **1**. Summary of the origin of the respective stable isotope labels and their final position in **1**.

With l‐alanine/pyruvate and previously l‐lysine determined as principal building blocks of **1** (Figure 6), we investigated whether the muscarine‐like alkaloids **3** and **4** [15] show heavy‐isotope incorporation as well, when supplied with U‐^13^C^15^N‐l‐lysine and 2,3‐^13^C_2_‐l‐alanine. The incorporation of these two amino acids was evident from mass spectrometric analyses, which showed the increased *m*/*z* values (Figure S7). Hence, these data strongly suggest that **3** und **4** originate from the same biosynthetic pathway as **1**.

Our revised model of **1** biosynthesis allows to infer biosynthetic enzymes. Obvious steps include the initial SAM‐dependent trimethylation of l‐lysine by a methyltransferase and the presumably ATP‐dependent phosphotransfer to **1**, yielding **3**, by a kinase as a late step. Some intermediate steps must remain rather elusive, in particular the coupling reaction which establishes the **1** backbone. Yet, assuming **9** is processed so that the Cβ can act as an electron donor, coupling could proceed with pyruvate as the electron acceptor, catalyzed by an aldolase (Figure S6). Alternatively, **9** may be processed so that the Cβ can act as an electron acceptor. In this scenario, coupling with l‐alanine could require its prior pyridoxal 5’‐phosphate (PLP)‐dependent oxidative decarboxylation. Should pyruvate, rather than l‐alanine, serve as coupling partner, thiamine‐dependent pyruvate decarboxylase activity would be required.

A major further processing step is the required cleavage of the Cα‐Cβ bond, which could be carried out by PLP‐dependent α‐deamination of **9**, allowing later for a possible cleavage through a Baeyer–Villiger oxidation, catalyzed by a flavine‐dependent oxygenase, and subsequent hydrolysis of the ester to remove the excess C_2_ fragment, possibly liberating **4**. The presumably last major step is the formation of the tetrahydrofurane, likely by oxidative cyclization. For a more profound presentation of possible reactions and enzymes, please refer to Figure S6.

## 3 Conclusion

In summary, our results revise the established biosynthesis model of **1**. We provide unequivocal evidence for l‐alanine and l‐lysine as building blocks of the carbon/nitrogen backbone of the quaternary ammonium natural product **1** (Figure 6). Furthermore, SAM‐dependent triple methyltransfer to l‐lysine, yielding **9**, initiates the biosynthesis, thereby establishing a quaternary ammonium compound. Such natural products play essential roles for biological systems. Among the most prominent examples is **7**, highlighted in this article as well, which furthermore functions as an indispensable cellular fatty acid carrier [21]. As second example, **2** serves as an important neurotransmitter with which **1** interferes as a toxin [5, 6]. Still, the true function of **1** for the producing organisms remains to be elucidated. Besides **1**, another case of a mushroom quaternary ammonium natural product includes aeruginascin, which is related to the hallucinogenic psilocybin [27].

Together with **5** and psilocybin, the fatal toxin **1** represents the prime example of a mushroom natural product that mimic endogenous ligands and exerts pharmacologically extremely potent effects on the human nervous system. Intriguingly, all three compounds do not require complex amino acid oligomerization and macrocyclization steps familiar from countless other microbial pathways and involving nonribosomal peptide synthetases or ribosomally made and posttranslationally modified peptides.

Rather, a single amino acid is coupled either with a second one or with an α‐keto acid to contribute the atoms of the molecule's backbone. In the case of psilocybin and **5**, only a single amino acid is involved. Even though these compounds do not reach the degree of complexity of many other natural products, new and potentially pharmacologically relevant congeners await discovery, as shown by **3**, or norpsilocin which is a neuroactive psilocybin shunt product [28, 29]. We conclude that the more profound insight into **1** biosynthesis from this work will eventually help discover as yet unknown metabolic intermediates that may represent powerful mushroom toxins as well.

## Conflicts of Interest

The authors declare no conflicts of interest.

## Supporting information




**Supporting File**: anie72234‐sup‐0001‐SuppMat.pdf.
The authors have cited additional references within the Supporting Information [30, 31, 32, 33, 34, 35, 36, 37, 38, 39, 40, 41, 42, 43, 44, 45, 46, 47, 48, 49, 50, 51, 52, 53, 54, 55].

## Data Availability

The data that supports the findings of this study are available in the supplementary material of this article.

## References

[1] S. Govorushko , R. Rezaee , J. Dumanov , and A. Tsatsakis , “Poisoning Associated With the Use of Mushrooms: A Review of the Global Pattern and Main Characteristics,” Food and Chemical Toxicology 128 (2019): 267–279, 10.1016/j.fct.2019.04.016. 30995515

[2] M.‐Q. He , M.‐Q. Wang , Z.‐H. Chen , et al., “Potential Benefits and Harms: A Review of Poisonous Mushrooms in the World,” Fungal Biology Reviews 42 (2022): 56–68, 10.1016/j.fbr.2022.06.002.

[3] S. Parnmen , N. Nooron , S. Leudang , et al., “Foodborne Illness Caused by Muscarine‐containing Mushrooms and Identification of Mushroom Remnants Using Phylogenetics and LC‐MS/MS,” Food Control 128 (2021): 108182, 10.1016/j.foodcont.2021.108182.

[4] K. M. Schenk‐Jaeger , C. Rauber‐Lüthy , M. Bodmer , H. Kupferschmidt , G. A. Kullak‐Ublick , and A. Ceschi , “Mushroom Poisoning: A Study on Circumstances of Exposure and Patterns of Toxicity,” European Journal of Internal Medicine 23 (2012): e85–e91, 10.1016/j.ejim.2012.03.014. 22560399

[5] A. S. V. Burgen , “History and Basic Properties of the Muscarinic Cholinergic Receptor,” The Muscarinic Receptors, ed. J. H. Brown , (Humana Press, 1989), 3–27, 10.1007/978-1-4612-4498-1.

[6] A. C. Kruse , B. K. Kobilka , D. Gautam , P. M. Sexton , A. Christopoulos , and J. Wess , “Muscarinic Acetylcholine Receptors: Novel Opportunities for Drug Development,” Nature Reviews Drug Discovery 13 (2014): 549–560, 10.1038/nrd4295. 24903776 PMC5818261

[7] S. J. Hill , “G‐protein‐Coupled Receptors: Past, Present and Future,” British Journal of Pharmacol *og* *y* 147 (2006): S27–37, 10.1038/sj.bjp.0706455. PMC1760739 16402114

[8] P. Montuschi and G. Ciabattoni , “Bronchodilating Drugs for Chronic Obstructive Pulmonary Disease: Current Status and Future Trends,” Journal of Medicinal Chemistry 58 (2015): 4131–4164, 10.1021/jm5013227. 25587755

[9] M. C. Michel , “Fesoterodine: A Novel Muscarinic Receptor Antagonist for the Treatment of Overactive Bladder Syndrome,” Expert Opinion on Pharmacotherapy 9 (2008): 1787–1796, 10.1517/14656566.9.10.1787. 18570610

[10] S. E. Yohn , P. J. Weiden , C. C. Felder , and S. M. Stahl , “Muscarinic Acetylcholine Receptors for Psychotic Disorders: Bench‐side to Clinic,” Trends in Pharmacological Sciences 43 (2022): 1098–1112, 10.1016/j.tips.2022.09.006. 36273943

[11] O. Schmiedeberg and R. Koppe , Das Muscarin: Das Giftige Alkaloid Des Fliegenpilzes (Agaricus Muscarius L.) (Verlag von F.C.W. Vogel, 1869), 1–111.

[12] F. Kögl , C. A. Salemink , H. Schouten , and F. Jellinek , “Über Muscarin. III,” Recueil des Travaux Chimiques des Pays‐Bas 76 (1957): 109–127, 10.1002/recl.19570760204.

[13] V. Rodríguez‐Tzompanzi , L. Quintero , D. M. Tepox‐Luna , S. Cruz‐Gregorio , and F. Sartillo‐Piscil , “Blue light photoredox decarboxylation and tin‐free Barton‐McCombie reactions in the stereoselective synthesis of (+)‐muscarine,” Tetrahedron Letters 60 (2019): 423–426, 10.1016/j.tetlet.2018.12.058.

[14] A. Gehlawat , R. Prakash , and S. Kumar Pandey , “A Short and Efficient Enantioselective Synthesis of (+)‐(2S ,3S ,5S)‐Epi‐Muscarine,” ChemistrySelect 5 (2020): 6373–6375, 10.1002/slct.202001598.

[15] S. Dörner , F. Trottmann , P. M. Jordan , et al., “The Fatal Mushroom Neurotoxin Muscarine is Released from a Harmless Phosphorylated Precursor upon Cellular Injury,” Angewandte Chemie International Edition 63 (2024): e202417220, 10.1002/anie.202417220. 39432715

[16] P. Kosentka , S. L. Sprague , M. Ryberg , et al., “Evolution of the Toxins Muscarine and Psilocybin in a Family of Mushroom‐Forming Fungi,” PLoS ONE 8 (2013): e64646, 10.1371/journal.pone.0064646. 23717644 PMC3662758

[17] R. J. Stadelmann , C. H. Eugster , and E. Müller , “Über die Verbreitung der Stereomeren Muscarine Innerhalb der Ordnung der Agaricales,” Helvetica Chimica Acta 59 (1976): 2432–2436, 10.1002/hlca.19760590718. 1017968

[18] K. Nitta , R. J. Stadelmann , and C. H. Eugster , “Zur Biogenese Des Muscarins in Mycelkulturen von *Clitocybe rivulosa* ,” Helvetica Chimica Acta 60 (1977): 1747–1753, 10.1002/hlca.19770600529. 893124

[19] S. Obermaier and M. Müller , “Ibotenic Acid Biosynthesis in the Fly Agaric Is Initiated by Glutamate Hydroxylation,” Angewandte Chemie International Edition 59 (2020): 12432–12435, 10.1002/anie.202001870. 32233056 PMC7383597

[20] S. Obermaier , (2019), “Biocatalytic Phenol Coupling of γ‐Naphthopyrones and Biosynthesis of Ibotenic Acid,” PhD thesis, Albert‐Ludwigs‐Universität.

[21] S. Rippa , Y. Zhao , F. Merlier , A. Charrier , and Y. Perrin , “The Carnitine Biosynthetic Pathway in *Arabidopsis thaliana* Shares Similar Features With the Pathway of Mammals and Fungi,” Plant Physiology and Biochemistry 60 (2012): 109–114, 10.1016/j.plaphy.2012.08.001. 22922110

[22] Z.‐M. He , Z.‐H. Chen , T. Bau , G.‐S. Wang , and Z. L. Yang , “Systematic Arrangement Within the family Clitocybaceae (Tricholomatineae, Agaricales): Phylogenetic and Phylogenomic Evidence, Morphological Data and Muscarine‐producing Innovation,” Fungal Diversity 123 (2023): 1–47, 10.1007/s13225-023-00527-2.

[23] A. Vit , L. Misson , W. Blankenfeldt , and F. P. Seebeck , “Ergothioneine Biosynthetic Methyltransferase EgtD Reveals the Structural Basis of Aromatic Amino Acid Betaine Biosynthesis,” ChemBioChem 16 (2015): 119–125, 10.1002/cbic.201402522. 25404173

[24] A. Kazaks , M. Makrecka‐Kuka , J. Kuka , et al., “Expression and Purification of Active, Stabilized Trimethyllysine Hydroxylase,” Protein Expression and Purification 104 (2014): 1–6, 10.1016/j.pep.2014.09.002. 25220864

[25] A. H. K. Al Temimi , B. J. G. E. Pieters , Y. V. Reddy , P. B. White , and J. Mecinović , “Substrate Scope for Trimethyllysine Hydroxylase Catalysis,” Chemical Communications 52 (2016): 12849–12852, 10.1039/c6cc07845a. 27730239

[26] H. Xu , B. Andi , J. Qian , A. H. West , and P. F. Cook , “The α‐Aminoadipate Pathway for Lysine Biosynthesis in Fungi,” Cell Biochemistry and Biophysics 46 (2006): 43–64, 10.1385/CBB:46:1:43. 16943623

[27] N. Jensen , J. Gartz , and H. Laatsch , “Aeruginascin, a Trimethylammonium Analogue of Psilocybin From the Hallucinogenic Mushroom *Inocybe* *aeruginascens* ,” Planta Medica 72 (2006): 665–666, 10.1055/s-2006-931576. 16673333

[28] C. Lenz , J. Wick , and D. Hoffmeister , “Identification of ω‐ N ‐Methyl‐4‐hydroxytryptamine (Norpsilocin) as a *Psilocybe* Natural Product,” Journal of Natural Products 80 (2017): 2835–2838, 10.1021/acs.jnatprod.7b00407. 28929753

[29] A. M. Sherwood , A. L. Halberstadt , A. K. Klein , et al., “Synthesis and Biological Evaluation of Tryptamines Found in Hallucinogenic Mushrooms: Norbaeocystin, Baeocystin, Norpsilocin, and Aeruginascin,” Journal of Natural Products 83 (2020): 461–467, 10.1021/acs.jnatprod.9b01061. 32077284

[30] M. Moser , Die Pilze Mitteleuropas, 4^th^ ed. (Klinkhardt, 1960), 58–62.

[31] N. M. T. Lourenço , C. M. Monteiro , and C. A. M. Afonso , “Ionic Acylating Agents for the Enzymatic Resolution of sec‐Alcohols in Ionic Liquid,” European Journal of Organic Chemistry 2010 (2010): 6938–6943, 10.1002/ejoc.201000640.

[32] M. A. Gamal‐Eldin and D. H. Macartney , “Selective Molecular Recognition of Methylated Lysines and Arginines by Cucurbit[6]Uril and Cucurbit[7]Uril in Aqueous Solution,” Organic & Biomolecular Chemistry 11 (2013): 488–495, 10.1039/C2OB27007B. 23202694

[33] D. Zelencova‐Gopejenko , A. Grandane , E. Loza , et al., “Binding versus Enzymatic Processing of ε‐Trimethyllysine Dioxygenase Substrate Analogues,” Acs Medicinal Chemistry Letters 13 (2022): 1723–1729, 10.1021/acsmedchemlett.2c00261. 36385923 PMC9661700

[34] J. W. Lamppa , S. A. Tanyos , and K. E. Griswold , “Engineering *Escherichia coli* for Soluble Expression and Single Step Purification of Active Human Lysozyme,” Journal of Biotechnology 164 (2013): 1–8, 10.1016/j.jbiotec.2012.11.007. 23220215 PMC3594478

[35] Y. V. Reddy , A. H. K. Al Temimi , P. B. White , and J. Mecinović , “Evidence That Trimethyllysine Hydroxylase Catalyzes the Formation of (2S,3S)‐3‐Hydroxy‐Nε‐trimethyllysine,” Organic Letters 19 (2017): 400–403, 10.1021/acs.orglett.6b03608. 28045275

[36] M. Hassan , S. Morimoto , H. Murakami , T. Ichiyanagi , and N. Mori , “Purification and Characterization of 4‐ N ‐Trimethylamino‐1‐butanol Dehydrogenase of *Pseudomonas* sp. 13CM,” Bioscience, Biotechnology, and Biochemistry 71 (2007): 1439–1446, 10.1271/bbb.60510. 17587673

[37] G. R. Fulmer , A. J. Miller , N. H. Sherden , et al., “NMR Chemical Shifts of Trace Impurities: Common Laboratory Solvents, Organics, and Gases in Deuterated Solvents Relevant to the Organometallic Chemist,” Organometallics 29 (2010): 2176–2179, 10.1021/om100106e.

[38] KEGG pathway (lysine) accessible under: https://www.genome.jp/kegg‐bin/show_pathway?abp00300.

[39] KEGG pathway (carnitine) accessible under: https://www.genome.jp/kegg‐bin/show_pathway?abp00310.

[40] T. Laubach , A. Von Haeseler , and M. J. Lercher , “TreeSnatcher plus: Capturing Phylogenetic Trees From Images,” BMC Bioinformatics [Electronic Resource] 13 (2012): 110, 10.1186/1471-2105-13-110. 22624611 PMC3411374

[41] F. Trottmann , K. Ishida , J. Franke , et al., “Sulfonium Acids Loaded Onto an Unusual Thiotemplate Assembly Line Construct the Cyclopropanol Warhead of a *Burkholderia* Virulence Factor,” Angewandte Chemie International Edition 59 (2020): 13511–13515, 10.1002/anie.202003958. 32314848 PMC7496086

[42] D. A. Yee , T. B. Kakule , W. Cheng , et al., “Genome Mining of Alkaloidal Terpenoids From a Hybrid Terpene and Nonribosomal Peptide Biosynthetic Pathway,” Journal of the American Chemical Society 142 (2020): 710–714, 10.1021/jacs.9b13046. 31885262 PMC7000236

[43] W. Zhang , N. T. Forester , P. Chettri , et al., “Characterization of the Biosynthetic Gene Cluster for the Ribosomally Synthesized Cyclic Peptide Epichloëcyclins in *Epichloë* *festucae* ,” Journal of Agricultural and Food Chemistry 71 (2023): 13965–13978, 10.1021/acs.jafc.3c03073. 37704203 PMC10540207

[44] R. Kozakai , T. Ono , S. Hoshino , et al., “Acyltransferase That Catalyses the Condensation of Polyketide and Peptide Moieties of Goadvionin Hybrid Lipopeptides,” Nature Chemistry 12 (2020): 869–877, 10.1038/s41557-020-0508-2. 32719482

[45] H. Zhao , “Recent Advances in Enzymatic Carbon–carbon Bond Formation,” RSC Advances 14 (2024): 25932–25974, 10.1039/D4RA03885A. 39161440 PMC11331486

[46] B. Fu and E. P. Balskus , “Discovery of C‐C Bond‐Forming and Bond‐Breaking Radical Enzymes: Enabling Transformations for Metabolic Engineering,” Current Opinion in Biotechnology 65 (2020): 94–101, 10.1016/j.copbio.2020.02.003. 32171888 PMC7670169

[47] G. Z. Dai , W. B. Han , Y. N. Mei , et al., “Pyridoxal‐5′‐phosphate–dependent Bifunctional Enzyme Catalyzed Biosynthesis of Indolizidine Alkaloids in Fungi,” Proceedings National Academy of Science USA 117 (2019): 1174–1180, 10.1073/pnas.1914777117. PMC6969511 31882449

[48] Y.‐L. Du and K. S. Ryan , “Pyridoxal Phosphate‐Dependent Reactions in the Biosynthesis of Natural Products,” Natural Product Reports 36 (2019): 430–457, 10.1039/C8NP00049B. 30183796

[49] M. Brovetto , D. Gamenara , P. Saenz Méndez , and G. A. Seoane , “C−C Bond‐Forming Lyases in Organic Synthesis,” Chemical Reviews 111 (2011): 4346–4403, 10.1021/cr100299p. 21417217

[50] G. de Gonzalo , M. D. Mihovilovic , and M. W. Fraaije , “Recent Developments in the Application of Baeyer–Villiger Monooxygenases as Biocatalysts,” ChemBioChem 11 (2010): 2208–2231, 10.1002/cbic.201000395. 20936617

[51] H. Leisch , K. Morley , and P. C. K. Lau , “Baeyer−Villiger Monooxygenases: More than Just Green Chemistry,” Chemical Reviews 111 (2011): 4165–4222, 10.1021/cr1003437. 21542563

[52] D. E. Torres Pazmiño , H. M. Dudek , and M. W. Fraaije , “Baeyer–Villiger Monooxygenases: Recent Advances and Future Challenges,” Current Opinion in Chemical Biology 14 (2010): 138–144, 10.1016/j.cbpa.2009.11.017. 20015679

[53] M. Richter , N. Traitcheva , U. Knüpfer , and C. Hertweck , “Sequential Asymmetric Polyketide Heterocyclization Catalyzed by a Single Cytochrome P450 Monooxygenase (AurH),” Angewandte Chemie International Edition 47 (2008): 8872–8875, 10.1002/anie.200803714. 18855960

[54] J. D. Rudolf , L. Dong , X. Zhang , H. Renata , and B. Shen , “Cytochrome P450‐Catalyzed Hydroxylation Initiating Ether Formation in Platensimycin Biosynthesis,” Journal of the American Chemical Society 140 (2018): 12349–12353, 10.1021/jacs.8b08012. 30216060 PMC6211292

[55] J. Pan , M. Bhardwaj , B. Zhang , et al., “Installation of the Ether Bridge of Lolines by the Iron‐ and 2‐Oxoglutarate‐Dependent Oxygenase, LolO: Regio‐ and Stereochemistry of Sequential Hydroxylation and Oxacyclization Reactions,” Biochemistry 57 (2018): 2074–2083, 10.1021/acs.biochem.8b00157, Jr. 29537853 PMC5895980

