# Supplementary material for: A Revised Model for Muscarine Biosynthesis Involving Lysine Trimethylation
Source: Angew Chem Int Ed Engl. 2026 Apr 23;65(24):e7581705. doi: 10.1002/anie.7581705 (PMC13245600; doi:10.1002/anie.7581705)
Supplement: Supplementary file 1 — Supporting File: anie72234‐sup‐0001‐SuppMat.pdf. The authors have cited additional references within the Supporting Information [30, 31, 32, 33, 34, 35, 36, 37, 38, 39, 40, 41, 42, 43, 44, 45, 46, 47, 48, 49, 50, 51, 52, 53, 54, 55]. [file ANIE-65-e7581705-s001.pdf]

Table of Contents

Experimental Section 2

Scheme S1. Detailed sequences of previous hypothetical pathways towards muscarine (**1**). 13
Figure S1. Simplified phylogenetic tree of the *Clitocybaceae* family. 15
Figure S2. Correlation of muscarine (**1**) biosynthesis and the capacity to produce trimethyl-L-lysine (**9**)............16
Figure S3. Biosynthetic divergence of the **1** and L-carnitine (**7**) pathways. 17
Figure S4. Full pyruvate stable-isotope labeling to identify the origin of carbon atoms C2 and 2-CH_3 in **1**. 18
Figure S5. MS/MS spectra and assigned fragments to trace stable isotope incorporation into **1**.........................19
Figure S6. Proposed biosynthetic pathways for **1**. 20
Figure S7. Mass spectrometric analysis of 4´-phosphomuscarine (**3**) and muscaridine (**4**)................................22
Table S1. Incorporation of the C_2 fragment into muscarine (**1**).. 23
Table S2. Incorporation of stable isotope-labeled atoms into **1**, traced by MS/MS. 24
Table S3. Fungal species used during this study. 26
Table S4. Chromatographic methods and instrumentation. 27

References. 29

Experimental Section

Chemicals: Reagents and components for microbiological media were purchased from Carl Roth, MedChem Express, Merck, Sigma-Aldrich, Thermo Fisher Scientific, and VWR. Solvents and **1** were purchased from Sigma-Aldrich, deuterated solvents were from CortecNet.

Fungal Strains. For fungal strains used during this study, please refer to Table S3. Strains were routinely maintained at 4 °C on Moser B[30] or on malt extract peptone (MEP) solid media (15 g L^{-1} malt extract, 3 g L^{-1} peptone, 18 g L^{-1} agar) and transferred to fresh MEP agar plates for this study.

Stable isotope labeling in *Collybia rivulosa*. *C. rivulosa* was precultured on MEP solid medium for 4-6 weeks and at 25 °C in the dark. For stable isotope labeling, mycelium-covered agar plugs (from actively growing mycelium, 5 mm diameter) were placed into a 24-well plate containing 1 mL MEP liquid medium per well, as well as 1 mM or 5 mM (for pyruvate, acetate, L-alanine, and L-alanine/L-lysine) co-feeding of the investigated compound (stable isotope labeled or non-labeled). The cultures were shaken at 130 rpm for 12 d at 25 °C in the dark. Subsequently, the mycelium was removed from the culture broth and frozen at -80 °C, and freeze dried. Then, the mycelium was pulverized with a spatula and extracted with MeOH (200 µL per culture) for 5 min by sonication, centrifuged (20,000 g for 1 min). The supernatant (100 µL) was subjected to mass spectrometry. Method I (Table S4) was used to analyze L-(+)-muscarine (**1**), L-carnitine (**7**), and muscaridine (**4**); method II (Table S4) was applied to analyze 4´-phosphomuscarine (**3**).

Biotransformation. The metabolic capacity of various fungal species investigated species within and outside of the family Clitocybaceae to biotransform ε-*N*,*N*-dimethyl-L-lysine (**10**) into ε-*N*,*N*,*N*-trimethyl-L-lysine (**9**) was tested. To this end, a cultivation procedure as described above for *C. rivulosa* was followed. Cultures were supplemented with 5 mM D_2-**10**. Depending on the growth rate of the respective species, the cultivation proceeded between 7 and 21 d. The extraction followed an identical procedure as described above for *C. rivulosa*. Method II (Table S4) was used to identify D_3-**9** in the respective extracts.

Chemical and biocatalytic syntheses. Stable isotope-labeled and non-labeled substrates were prepared either by organic synthesis or by combining enzymatic and synthetic reactions.

Synthesis of D_9-γ-butyrobetaine (**8**)

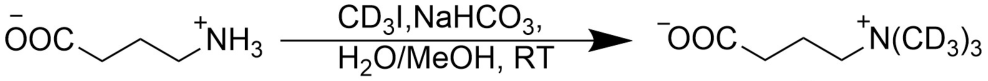

8

To synthesize D_9-**8**, 21 mg γ-aminobutyric acid (0.2 mmol, 1 eq.) were dissolved in 2 mL H_2O/MeOH (1:1) + $NaCO_3H$ (80 mg,1 mmol, 5 eq.) + 47 µL CD_3I (107 mg, 0.74 mmol, 3.7 eq.) and stirred overnight. The solvent was removed under reduced pressure, the residue was dissolved in water. Method III (Table S4) was used for purification. The organic solvent was removed under reduced pressure, and water was removed by lyophilization.

Yield: 20 mg, 0.13 mmol, 65%.

Analytical data of synthetic D_9-**8**:

^{1}H NMR (500 MHz, DMSO-d_6, ppm) δ = 3.20–3.25 (m, 2H), 2.13 (t, J = 6.9 Hz, 2H), 1.80–1.89 (m, 2H). The ^{1}H chemical shifts are in accordance with reported data for **8.**[31] The signal at δ = 3.04 (s, 9H, ($N(CH_3)_3$) is missing due to deuteration.

HRESIMS *m*/*z* 155.1740 (calcd. 155.1741 for $C_7H_7D_9NO_2^+$).

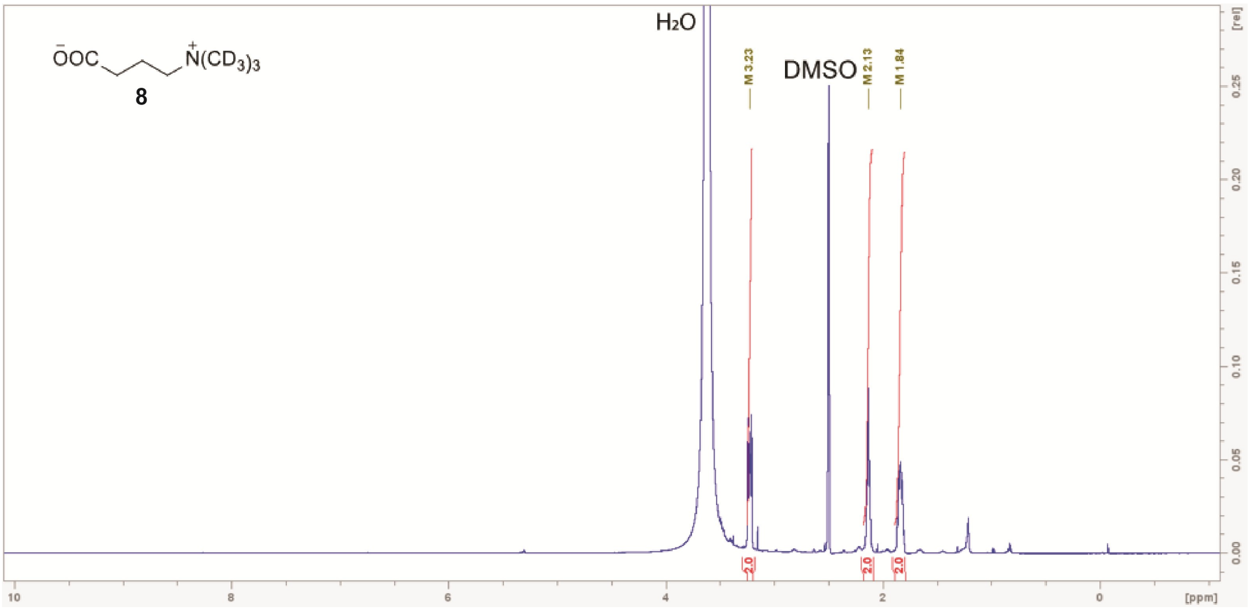

^{1}H NMR spectrum of D_9-γ-butyrobetaine (**8**) in DMSO-d_6.

Synthesis of ε-*N*,*N*-dimethyl-L-lysine (**10**) and D_2-**10**.

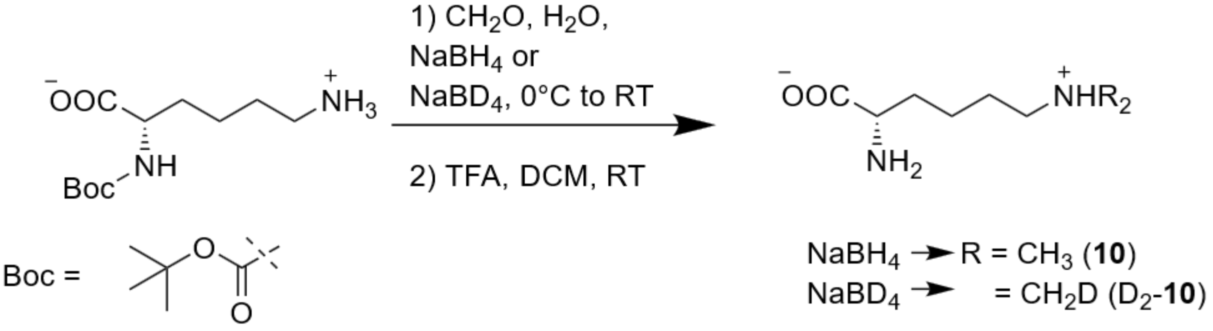

To a stirred solution of Boc-Lys-OH (0.5 mmol, 123 mg, 1 eq.) in 0.5 mL H_2O, 37% aqueous formaldehyde (157 µL, 2 mmol, 4 eq.) was added. After stirring at 0 °C for 5 min, 44 mg $NaBH_4$ or 52 mg $NaBD_4$ (1.25 mmol, 2.5 eq.) was added gradually. After one hour of stirring at room temperature, the solution was cooled again to 0 °C and the pH adjusted to 4-5 with 5 M HCl. Formaldehyde and $NaBH_4$ or $NaBD_4$ addition was repeated, and the solution was stirred for 1 h. Subsequently, $NaBH_4$ or $NaBD_4$ was quenched by adjusting the pH to 4-5, and the product was purified by method IV (Table S4). The organic solvent was removed under reduced pressure, and water was removed by lyophilization. The residue was dissolved in 5 mL dichloromethane (DCM), and 1 mL of trifluoroacetic acid (TFA) was added. The solution was stirred for 1 h at room temperature. The organic solvent was removed under reduced pressure and subsequent lyophilization.

Yield over two steps: 50 mg, 0.29 mmol, 58%.

Analytical data of synthetic **10** and D_2-**10**:

10: ^1H NMR (500 MHz, D_2O, ppm): δ = 3.92 (t, 1H, J = 6.3 Hz), 3.41 (m, 2H), 2.86 (s, 6H), 1.94 (m, 2H), 1.76 (m, 2H), 1.48 (m, 2H). The ^1H chemical shifts are in accordance with reported data for **10**.[32]

HRESIMS *m*/*z* 175.1442 (calcd. 175.1441 for $C_8H_{19}N_2O_2^+$).

D_2-**10:** ^1H NMR (500 MHz, D_2O, ppm): δ = 3.91 (t, 1H, J = 6.2 Hz), 3.16–3.07 (m, 2H), 2.82 (s, 4H), 1.97–1.86 (m, 2H), 1.78–1.70 (m, 2H), 1.53–1.37 (m, 2H). The ^1H chemical shifts are in accordance with reported data for **10**.[32], The signal at δ = 2.82 ppm (s) showed an intensity of 4H instead of 6H due to deuteration.

HRESIMS *m*/*z* 177.1567 (calcd. 177.1567 for $C_8H_{17}D_2N_2O_2^+$).

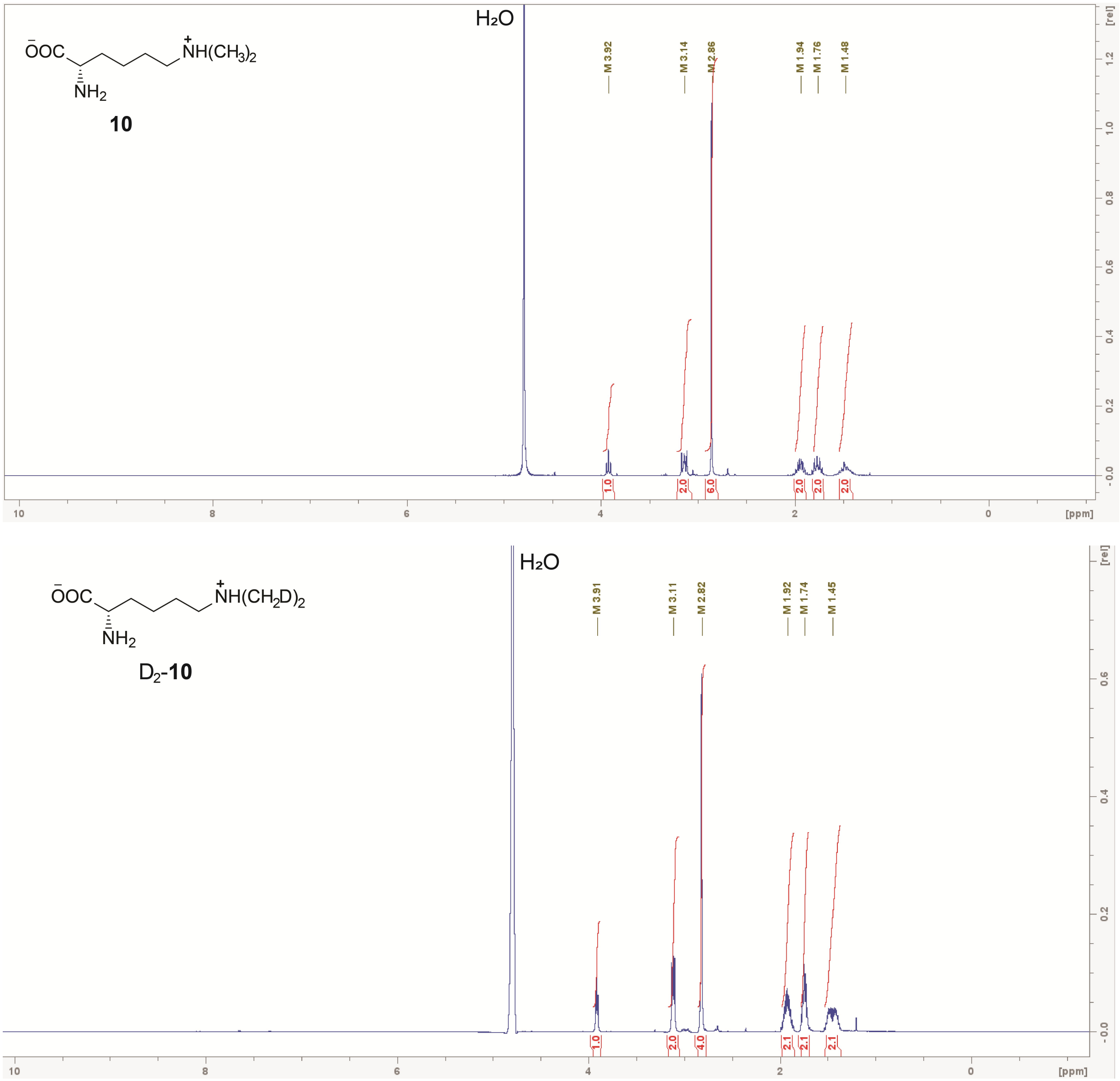

^{1}H NMR spectrum of ε-*N*,*N*-dimethyl-L-lysine (**10**) and D_2-**10** in D_2O.

Synthesis of ε-*N*,*N*,*N*-trimethyl-L-lysine (**9**) and D3-**9**.

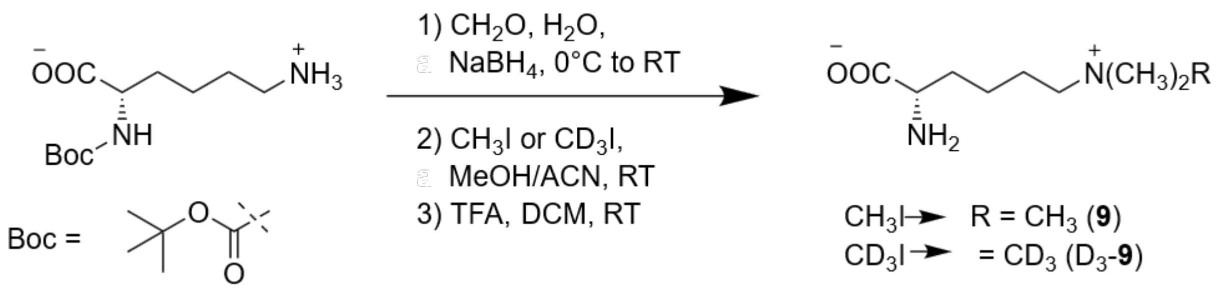

Boc-DML-OH was synthesized and purified from Boc-Lys-OH (0.5 mmol, 123 mg, 1 eq.) according to the procedure described in the synthesis of **10**. Boc-DML-OH (79 mg, 0.29 mmol, 0.58 eq.) was dissolved in ACN and H_2O (1 mL each). CH_3I (62 µL, 1 mmol, 2 Eq.) or CD_3I (62 µL, 1 mmol 2 Eq) was added. The reaction was stirred overnight. Afterwards Na_2CO_3 (106 mg, 1 mmol, 2 Eq.)) was added and again CH_3I (62 µL, 1 mmol, 2 Eq.) or CD_3I (62 µL, 1 mmol, 2 Eq.) and stirred overnight. After the reaction hat finished 5 mL of H_2O were added, and the product chromatographically purified using method IV (Table S4). The organic solvent was removed under reduced pressure, and water was removed by lyophilization. The residue was dissolved in 5 mL 1 M HCl. The solution was stirred overnight at room temperature. The solvent was by lyophilization.

Yield over three steps: 35 mg, 0.19 mmol, 38%.

Analytical data of synthetic **9** and D3-**9**:

9: ^{1}H NMR (300 MHz, D_2O, ppm): δ = 3.96 (t, J = 6.4 Hz, 1H), 3.37–3.27 (m, 2H), 3.09 (s, 9H), 2.05–1.91 (m, 2H), 1.90–1.77 (m, 2H), 1.56–1.36 (m, 2H). The ^{1}H chemical shifts are in accordance to reported data for **9**.[25]

HRESIMS *m*/*z* 189.1597 (calcd. 189.1598 for $C_9H_{21}O_2N_2^+$).

D3-**9**: ^{1}H NMR (300 MHz, D_2O, ppm): δ = 3.86 (t, J = 6.4 Hz, 1H), 3.36–3.27 (m, 2H), 3.09 (s, 6H), 2.00–1.89 (m, 2H), 1.89–1.77 (m, 2H), 1.35–1.56 (m, 2H). The ^{1}H and chemical shifts are in accordance to reported data for **9**.[25] The signal at δ = 3.09 ppm (s) showed an intensity of 6H instead of 9H due to deuteration.

HRESIMS *m*/*z* 192.1786 (calcd. 192.1787 for $C_9H_{18}D_3N_2O_2^+$).

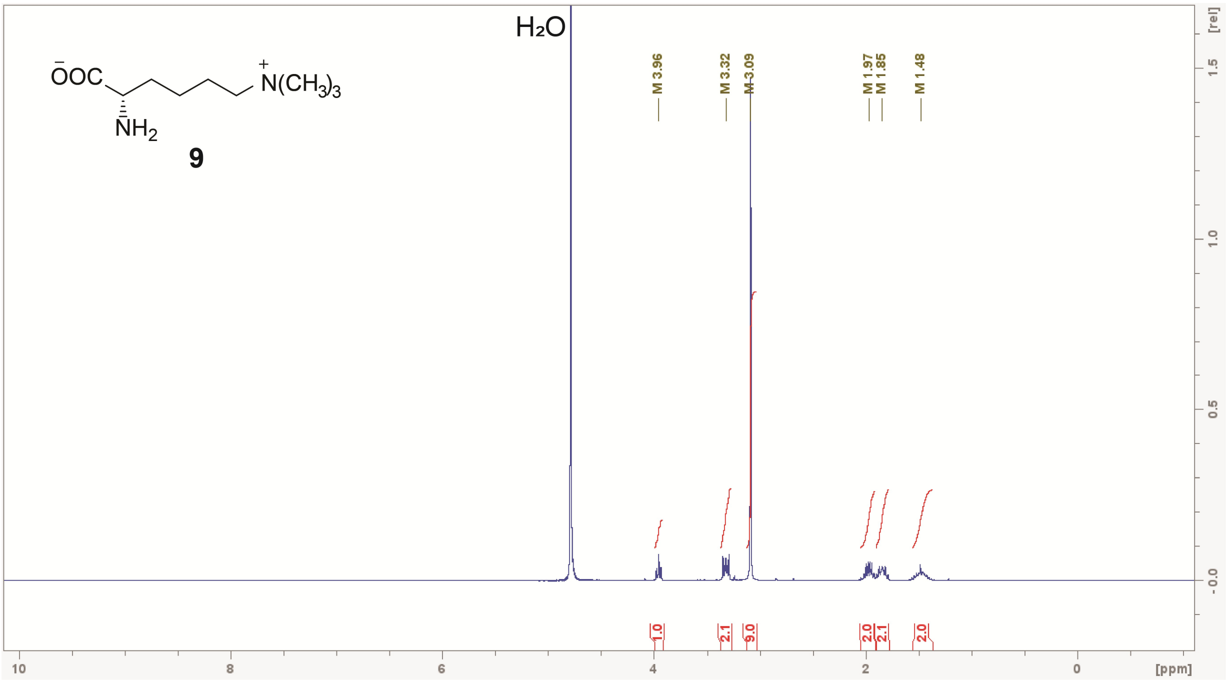

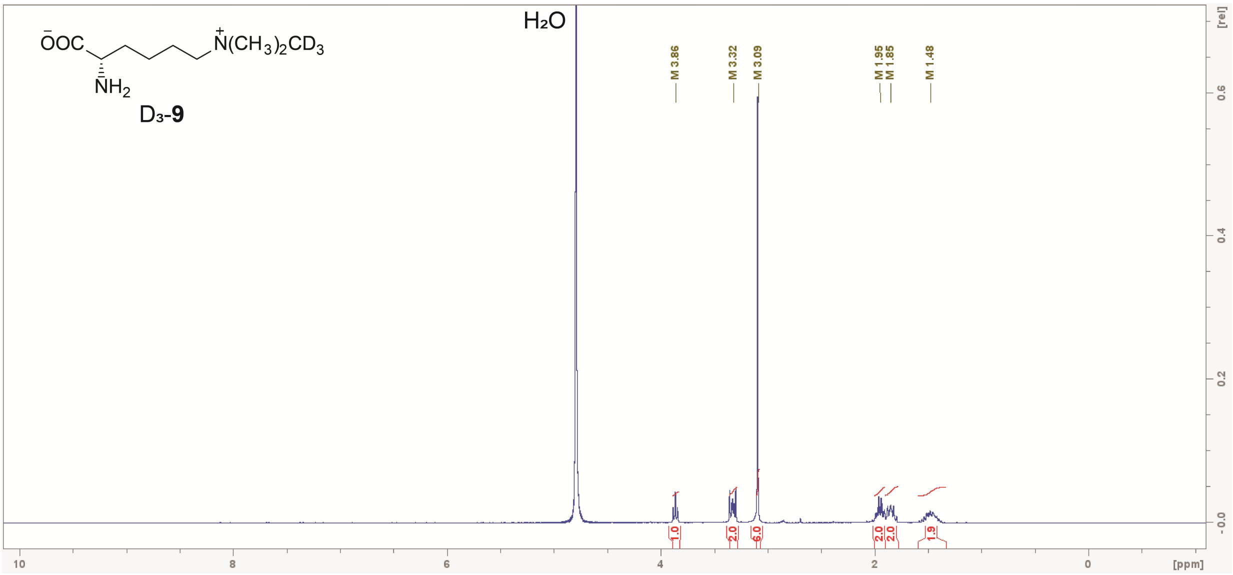

^{1}H NMR spectra of *ε-N,N,N*-trimethyl-L-lysine (**9**) and D_3-**9** in D_2O.

Synthesis of 3-hydroxy-ε-*N*,*N*,*N*-trimethyl-L-lysine (**11**) and D_3-**11**.

Production of trimethyllysine hydroxylase. A maltose binding protein (MBP) fusion protein of trimethyllysine hydroxylase (TMLHa, residues 40-417) was heterologously produced in *Escherichia coli* and purified, based on a slightly modified protocol described by Kazaks et al.[24] and Zelencova-Gopejenko et al.[33]

Competent *Escherichia coli* BL21 cells were co-transformed with a plasmid encoding a MBP-TMLHa fusion construct (kindly supplied by Dr. Diana Zelencova-Gopejenko[33], originally published by Kazaks *et al.* [24]) and pACYC-GroEL/ES-TF (Addgene plasmid #83923, the plasmid was deposited by Karl Griswold[34]).

The cells were cultivated in 2 × YT medium, supplemented with carbenicillin (100 µg mL^{-1}) and chloramphenicol (25 µg mL^{-1}), at 37 °C and 180 rpm until the OD_{600} reached 0.5. Subsequently, the culture was cooled down to 16 °C and induced by adding 0.1 mM isopropyl-β-D-thiogalactopyranosid. The incubation was continued at the same temperature and agitation for another 24 h. Afterwards, the cells were harvested by centrifugation at 4,000 × g and 4 °C, and the biomass was stored at −20 °C until further use. The cells were resuspended in ice-cold lysis buffer (20 mM Tris-HCl, pH 8.0, 200 mM NaCl, 0.7 M D-(+)-trehalose) and lysed by sonication. The supernatant was harvested by centrifugation at 4,000 × g for 50 min, filtered through a 0.45 µm filter, and purified using method VII (Table S4). The fractions containing MBP-TMLHa were combined and concentrated using an Amicon Ultra Centrifugal Filter Unit (50 kDa cut off, Merck Millipore), using a buffer composed of 50 mM HEPES, pH 7.4, and 150 mM NaCl. The enzyme concentration was determined photometrically using an AnalytikJena Scandrop UV-VIS spectrometer by measuring the absorbance at 280 nm, applying a theoretical molar extinction coefficient of 154,755 M^{-1} cm^{-1}.

Synthetic procedure for 3-hydroxy-ε-*N*,*N*,*N*-trimethyl-L-lysine (**11**) and D_3-**11**:

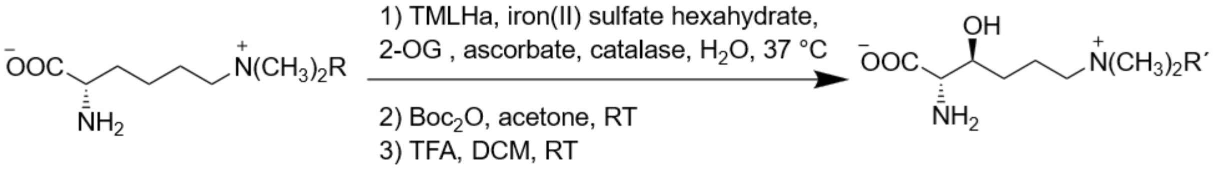

R = CH_3 (**9**)
= CD_3 (D_3-**9**)

R′ = CH_3 (**11**)
= CD_3 (D_3-**11**)

The enzyme-catalyzed reaction was carried out at 37 °C in a 27 mL buffer containing 50 mM HEPES pH 7.4 and 150 mM NaCl. To a pre-mixed and pre-warmed solution of **9** (4 mg, 500 µM), iron(II) sulfate hexahydrate (500 µM), 2-oxoglutarate (2-OG, 2.5 mM), ascorbate (5 mM), and catalase (v = 1 mg) was added TMLHa (1mL, 81 µM). After gentle stirring overnight at 37 °C, the pH was adjusted to 8, and Boc-anhydride (218,25 g·mol^{-1} 1.02 g·cm^{-3} 10 µL = 10 eq) and acetone 3 mL were added to the reaction mixture and vigorously stirred for 2 h.

Boc-OH-TML was purified using Method V (Table S4). The organic solvent was removed under reduced pressure, and water was removed by lyophilization. The residue was dissolved in 5 mL DCM and 1 mL of TFA was added. The solution was stirred for 1 h at room temperature. The organic solvent was removed under reduced pressure. The final purification of **11** was performed using method VI (Table S4). The organic solvent was removed under reduced pressure, and water was removed by lyophilization.

Yield over three steps: 2.1 mg, 0.24 mmol, 48.8 %.

Analytical data of synthetic **11** and D_3-**11**:

D_3-**11**: ^1H NMR (500 MHz, 0.1% DMSO in D_2O, ppm) δ = 4.14 (m, 1H), 3.86 (d, J = 3.2 Hz, 1H), 3.36 (m, 2H), 3.11 (s, 9H), 2.00 (m, 1H), 1.87 (m, 1H), 1.57 (m, 2H). The ^1H chemical shifts are in accordance with reported data for **11**.[35]

HRESIMS m/z 205.1553 (calcd. 205.1547 for $C_9H_{21}N_2O_3^+$).

D_3-**11**: ^1H NMR (600 MHz, 0.1% DMSO in D_2O, ppm) δ = 4.14 (m, 1H), 3.85 (d, J = 3.1 Hz, 1H), 3.35 (m, 2H), 3.11 (s, 6H), 2.00 (m, 1H), 1.88 (m, 1H), 1.57 (m, 2H). The ^1H chemical shifts are in accordance with reported data for **11**.[35] The signal at δ = 3.11 ppm (s) showed an integral of 6H instead of 9H due to deuteration.

HRESIMS m/z 208.1737 (calcd. 208.1735 for $C_9H_{18}D_3N_2O_3^+$).

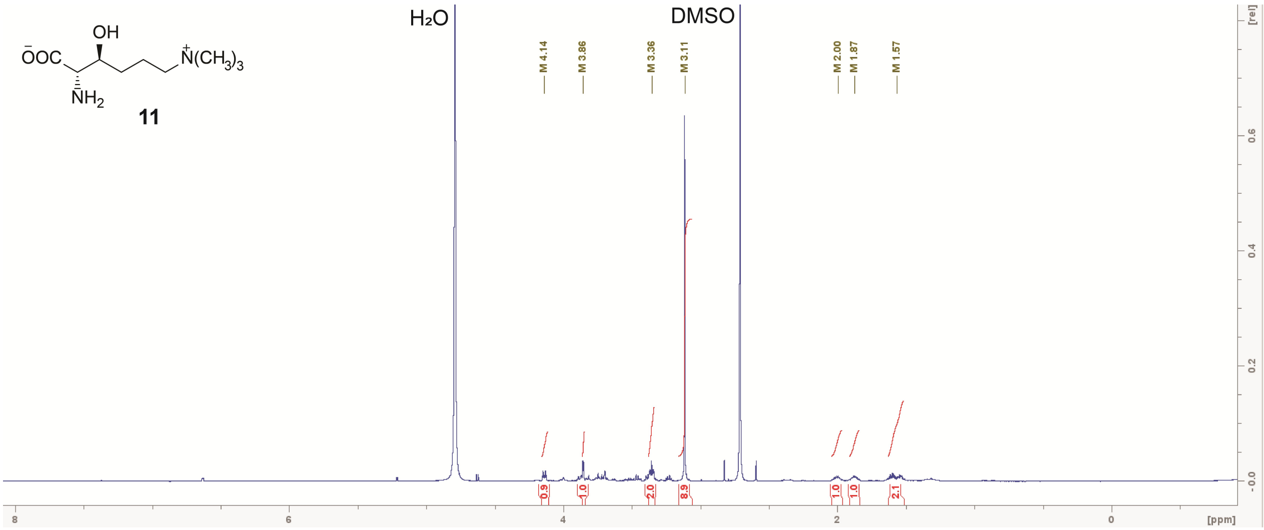

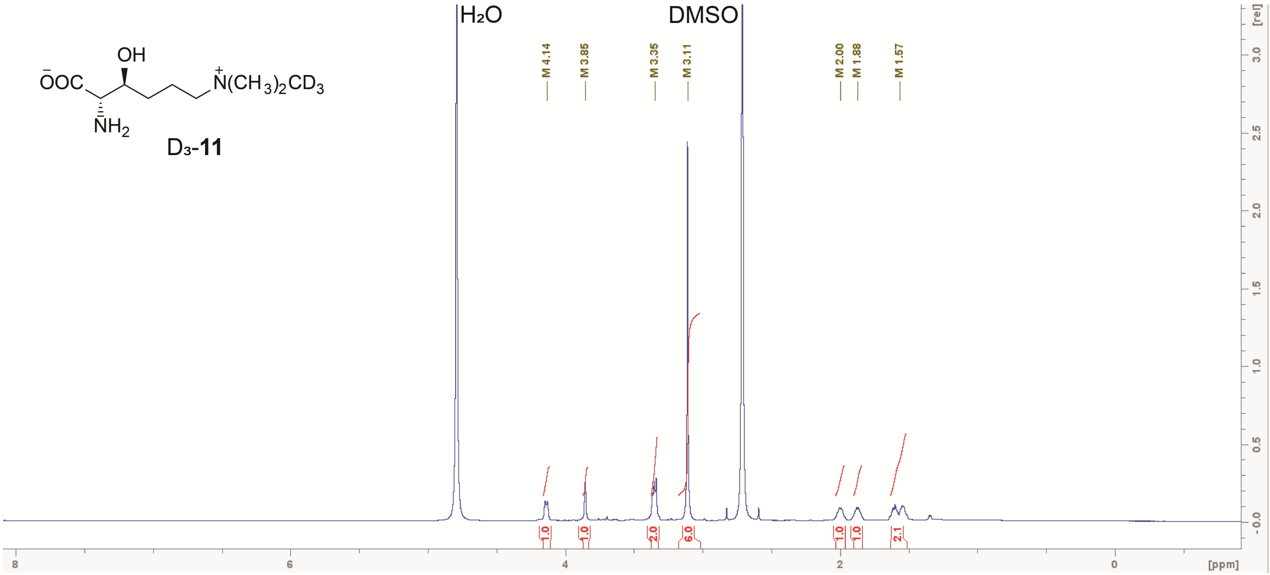

^1H NMR spectrum of 3-hydroxy-ε-N,N,N-trimethyl-L-lysine (**11**) in 0.1% and D_3-**11** in 0.1% DMSO in D_2O.

Synthesis of 4-(trimethylamino)butanal (**12**) iodide and D_3-**12** iodide.

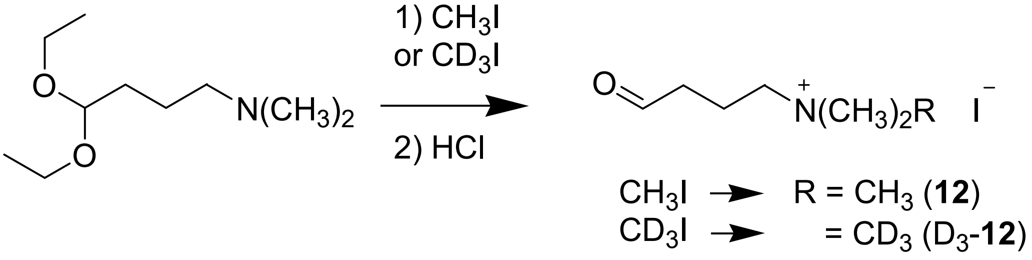

To a stirred solution of 4-(dimethylamino)butanal-dimethylacetal (0.5 mmol, 100 mg, 1 eq.) in 5 mL MeOH, iodomethane or D_3-iodomethane (50 µL, 0.8 mmol, 1,6 eq.) was added and stirred overnight. The organic solvent was removed under reduced pressure. The residue was dissolved in 4 mL Acetone and 1 mL H_2O and stirred under reflux for 3 hours. Subsequently 10 mL DCM was added and afterwards 10 mL H_2O. The aqueous phase was then further washed with 2 x 10 mL DCM. The aqueous phase was then frozen at -70 °C and lyophilized.

Yield over two steps: 123 mg, 0.48 mmol, 95.7%.

Analytical data of synthetic **12** and D_3-**12**:

12: ^{1}H NMR (600 MHz, D_2O, ppm) δ = 4.99 (t, J = 5.5 Hz, 1H), 3.26 (m, 2H), 3.01 (s, 9 H), 1.77 (m, 2H), 1.54 (m, 2H). The ^{1}H chemical shift of the aldehyde proton is in accordance to the reported literature **12**.[36] As the majority of **12** exists as the hydrate in D_2O the given shifts of the hydrate are stated.

HRESIMS *m*/*z* 130.1228 (calcd. 130.1226 for $C_7H_{16}NO^+$).

D_3-**12**: ^{1}H NMR (600 MHz, D_2O, ppm): 4.99 (t, J = 5.5 Hz, 1H), 3.26 (m, 2H), 3.01 (s, 6 H), 1.77 (m, 2H), 1.54 (m, 2H).

The ^{1}H chemical shift of the aldehyde proton is in accordance to the reported literature **12**.[36] As the majority of **12** exists as the hydrate in D_2O the given shifts of the hydrate are stated.

The signal at δ = 3.01 ppm (s) showed an integral of 6H, instead of 9H, due to deuteration.

HRESIMS *m*/*z* 133.1415 (calcd. 133.1415 for $C_7H_{13}D_3NO^+$).

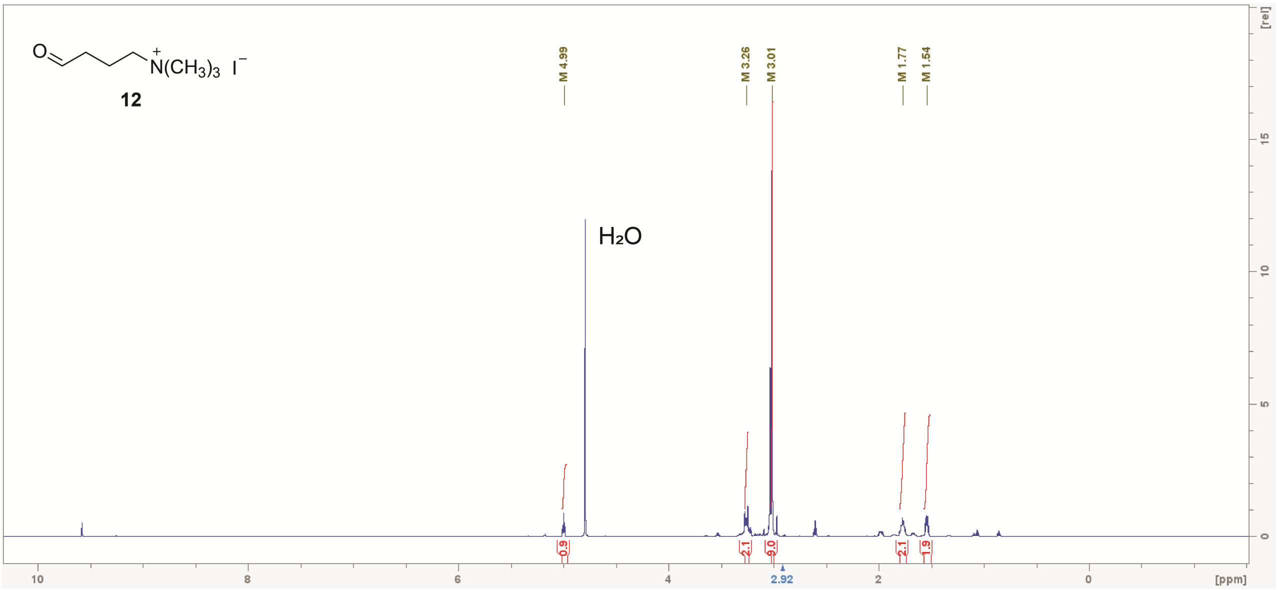

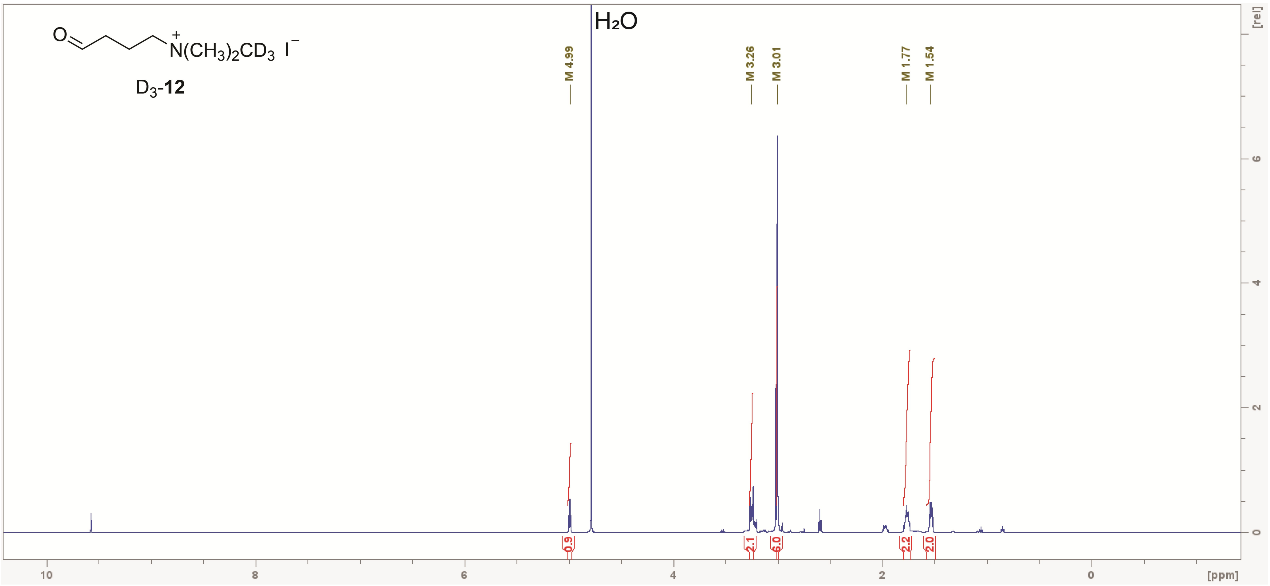

^{1}H NMR spectra of 4-(trimethylamino)butanal (**12**) and D_3-**12** in D_2O.

Liquid Chromatography. The following instruments were used (see also Table S4):

Instrument 1: Agilent 1290 Infinity II chromatograph, coupled with a 6130 quadrupole mass detector and operated with electrospray ionization. Detection was in positive mode.

Instrument 2: Thermo Fisher Scientific UltiMate 3000 UHPLC, coupled to a Q-Exactive HF-X Hybrid Quadrupole Orbitrap mass detector, also run with electrospray ionization.

Instrument 3: Agilent 1260 Infinity preparative chromatograph, equipped with a multi-wavelength detector.

Instrument 4: Agilent 1200 semi-preparative chromatograph, equipped with a diode array detector.

Instrument 5: GE Healthcare Äkta Pure 25.

For columns, gradients, and solvents, please refer to Table S4. Individual fractions from preparative and semi-preparative liquid chromatography were subsequently analyzed mass spectrometrically on instrument 2.

Nuclear Magnetic Resonance Spectroscopy. ^{1}H NMR spectra were recorded in deuterated solvents (D_2O or DMSO-d_6) at 298 K, using Bruker AVANCE II 300, AVANCE III 500 or 600 MHz spectrometers. The chemical shifts are reported in ppm, relative to the signal of residual non-deuterated solvent.[37] The following acronyms are used for multiplicities of resonance signals: s = singlet, d = doublet, t = triplet, q = quartet, qt = quintet, br = broad.

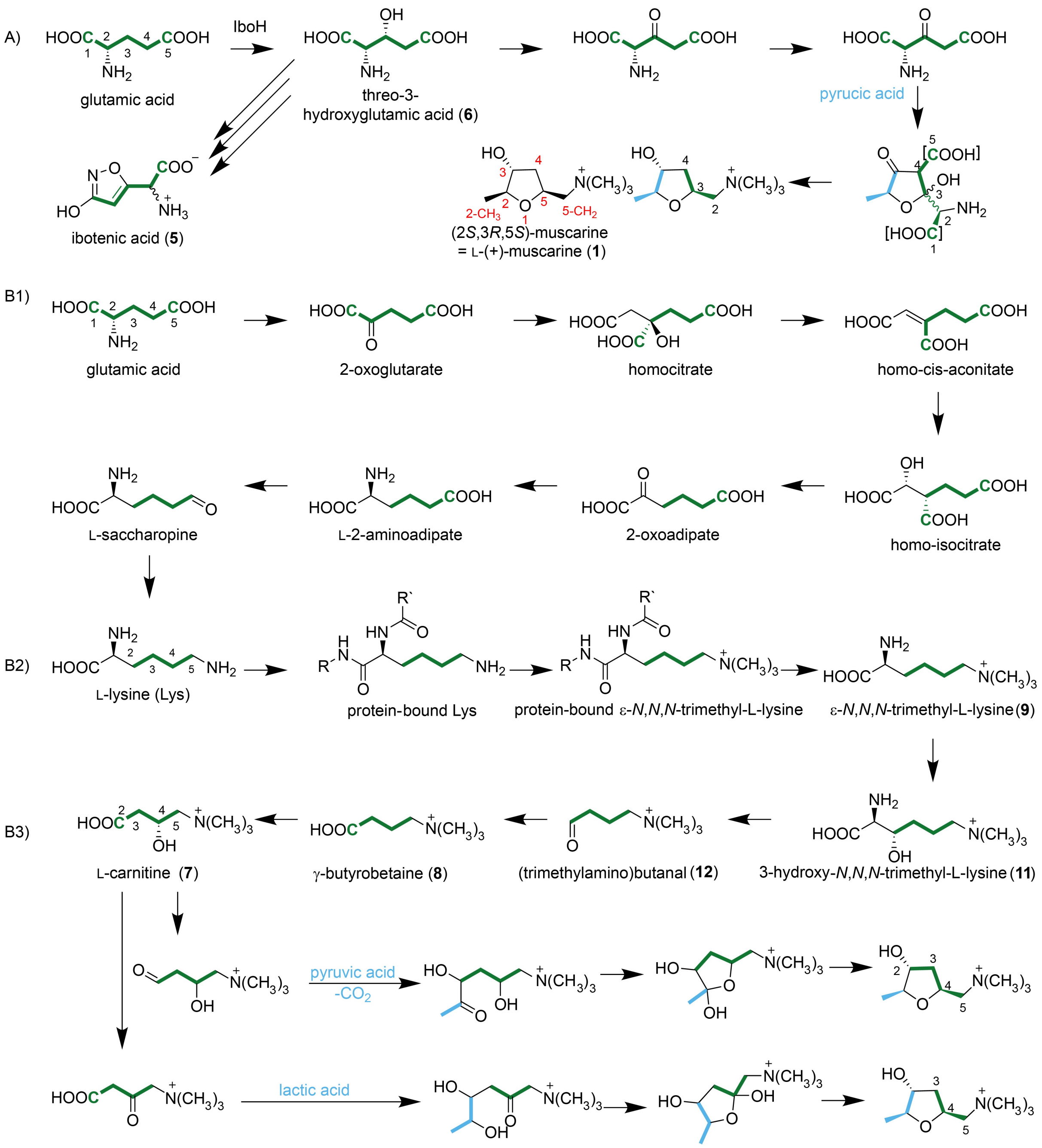

Scheme S1. Detailed sequences of previous hypothetical pathways towards muscarine and background information on previous hypotheses on muscarine biosynthesis

A) incorporation of L-glutamic acid into **1** and ibotenic acid, according to Nitta et al.[18] and the shown reaction catalyzed by IboH hydroxylase of the fly agaric mushroom *Amanita muscaria.*[19] Atom numbers of **1** are shown in red.

B) Hypothesis by Obermaier:[20] B1) incorporation of L-glutamic acid into L-lysine, B2) incorporation of L-lysine into L-carnitine (**7**), B3) proposed incorporation of **7** into L-(+)-muscarine (**1**). Black numbers represent the number of carbon atoms originating from L-glutamic acid.

Acronyms: OH-TML: 3-hydroxy-ε-*N*,*N*,*N*-trimethyl-L-lysine; TML: ε-*N*,*N*,*N*-trimethyl-L-lysine.

Background information

The only publication reporting experimental data to elucidate the biosynthesis of L-(+)-muscarine (**1**) was authored by Nitta et al.[18] The authors used various ^{14}C-labeled precursors and determined the incorporation based on the radioactivity of isolated **1** (Scheme S1 panel A). Subsequently, the position of incorporation was localized through chemical degradation of **1**. The authors concluded that L-glutamic acid (Glu) was the primary precursor, with C4, C5 and 5-CH_2 being derived from Glu C2-C4, which further suggests the **1** nitrogen atom originates from Glu. Radiotracer experiments with 1-^{14}C-Glu and 5-^{14}C Glu showed little incorporation, suggesting that these do not participate directly in **1** biosynthesis. Furthermore 2-CH_3, C2 and C3 were traced back to pyruvic acid and the origin of the *N*-methyl groups to formic acid and the "C1-pool". In addition, a common biosynthetic origin with ibotenic acid (**5**) was assumed, as both neuroactive compounds are produced by *A. muscaria* and both **1** and **5** were proposed to share the first intermediate, i.e., *threo*-3-hydroxyglutamic acid.

This particular stereoselective hydroxylation was shown to represent the first biosynthetic step in the biosynthesis of **5**, catalyzed by the 2-oxoglutarate-dependent dioxygenase IboH.[19] As genes encoding IboH-type dioxygenases were not found in other **1**-producing mushrooms,[20] a hypothesis was put forth more recently (Scheme S1, panel A) that assumes the conversion of Glu into L-lysine (Lys). Please see the respective KEGG pathway for details.[38] Subsequently, Lys is converted into L-carnitine (**7**) (see KEGG pathway and Scheme S1, panel B2)[39] which is then considered the starting point in the biosynthesis of **1** (Scheme S1, panel B3). Lactic or pyruvic acid are suggested to be the origin of the additional carbon atoms 2-CH_3, C2 and C3 of **1** that do not derive from **7**. However, this hypothesis conflicts with the observed incorporation of Glu by Nitta et al. as the carboxyl C5 of Glu is incorporated into **1** as 5-CH_2 (Scheme S1).

The radiotracer experiments to delineate **1** biosynthesis reported by Nitta et al.[18] need to be interpreted with some caution. Firstly, the absolute incorporation rate of ^{14}C-formic acid, reported for the N-methyl groups of **1,** is at least an order of magnitude higher than that of the other proposed incorporated precursors (75.5-fold higher than that of 1-^{14}C-pyruvic acid; 20-fold higher than that of 2-^{14}C-pyruvic acid; 25-fold higher than that of U-^{14}C-glutamic acid). Secondly, comparable absolute incorporation rates for pyruvic acid, labeled at different positions, are expected as the model proposed incorporation of all three carbon atoms of pyruvate into **1**. Yet, 2-^{14}C-pyruvic acid showed an absolute incorporation rate 3.8 times higher than 1-^{14}C-pyruvic acid. Furthermore, other tested compounds led to similarly high absolute incorporation rates than U-^{14}C-glutamic acid and 1-/2-^{14}C-pyruvic acid. Various reasons (or an unfortunate combination thereof) may have led to data misinterpretation. The reasons may include low incorporation rates, a relatively large instrument error, contamination during the reported sample preparation procedure, the lack of replicate measurements, and the general instrumental limitations half a century ago.

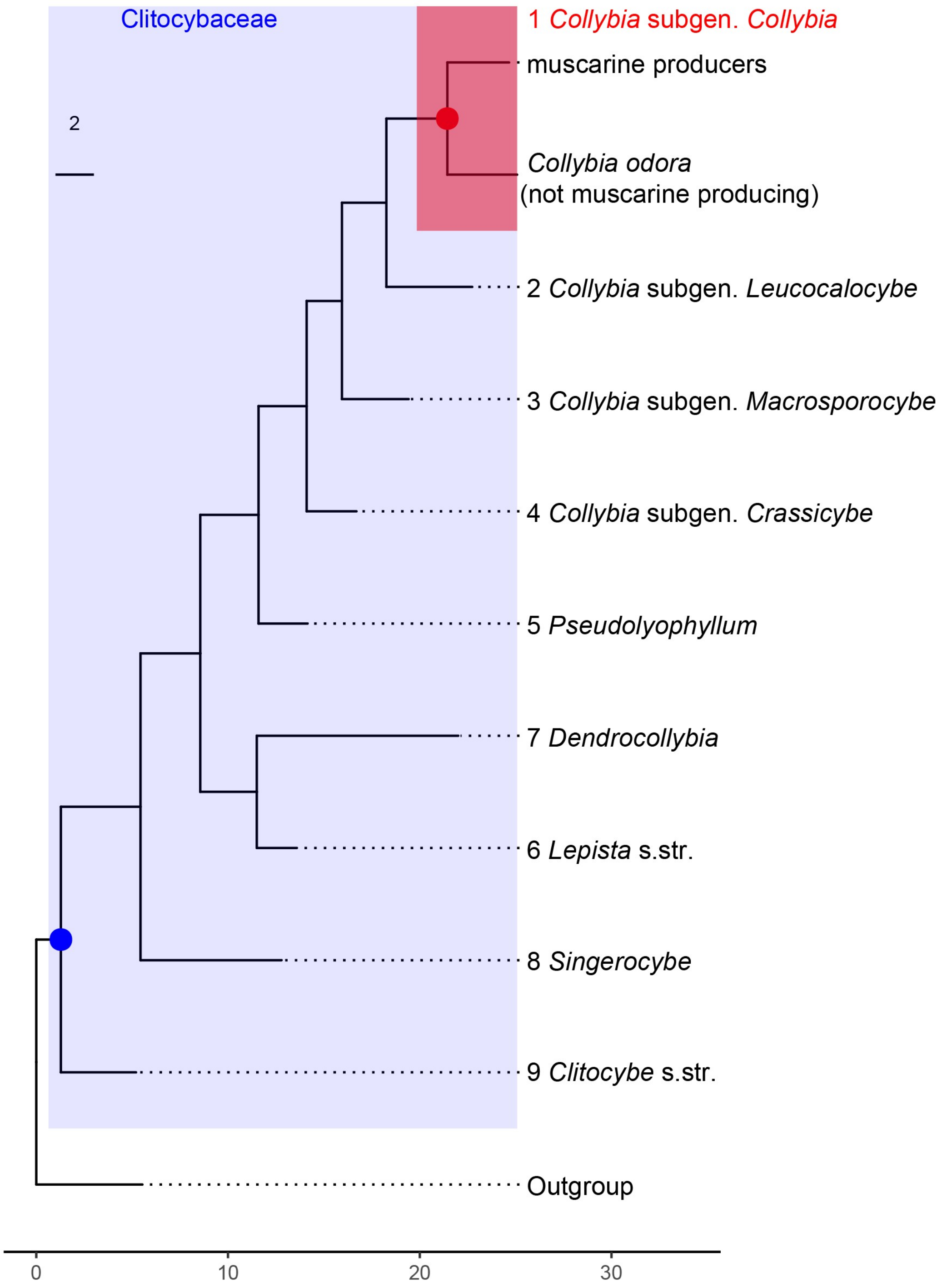

Figure S1. Simplified phylogenetic tree of the Clitocybaceae (funnel cap) family. This tree is based on the publication by He et al.[22] and was created using TreeSnatcher.[40] **1** producing species are restricted to clade 1 (shaded in red). However, the evolutionarily most ancient species in this subclade, *Collybia odora*, does not produce **1**.

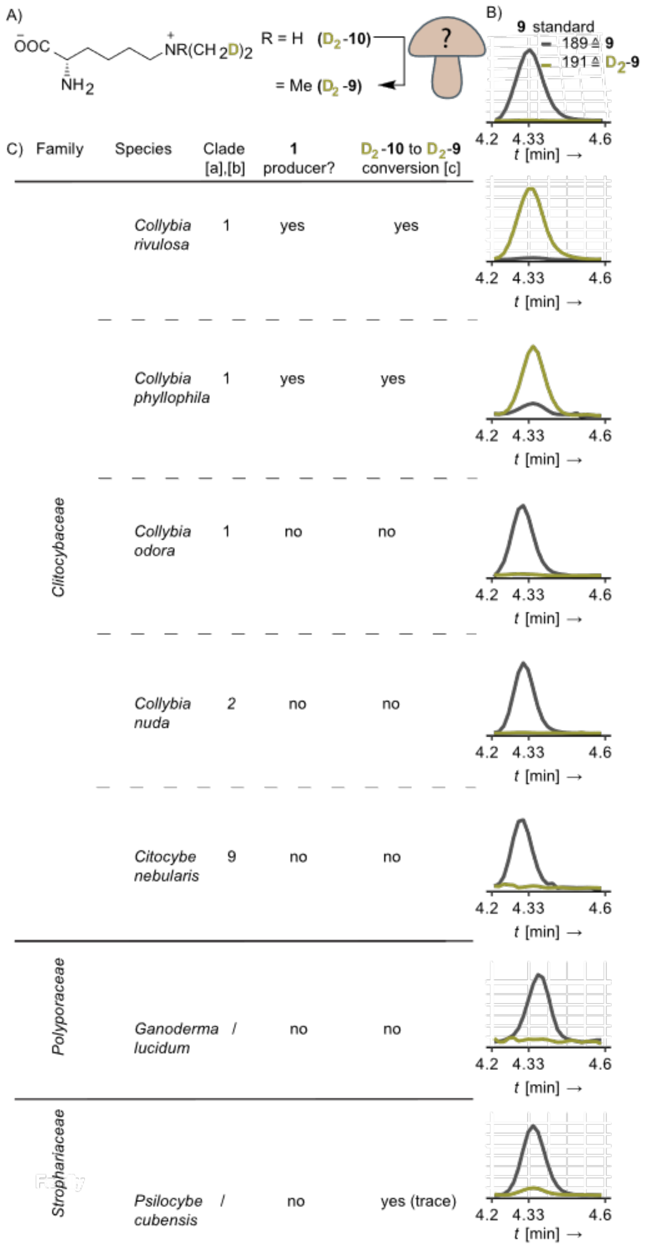

C) Family	Species	Clade [a],[b]	1 producer?	D_2-10 to D_2-9 conversion [c]
Clitocybaceae	*Collybia rivulosa*	1	yes	yes
	Collybia phyllophila	1	yes	yes
	Collybia odora	1	no	no
	Collybia nuda	2	no	no
	Citocybe nebularis	9	no	no
Polyporaceae	*Ganoderma lucidum*	/	no	no
Strophariaceae	*Psilocybe cubensis*	/	no	yes (trace)

Figure S2. Biosynthesis of **1** correlates with the capacity to produce **9**. A) The investigated reaction; B) EICs for **9** (*m*/*z* 189) and D_2-**9** (*m*/*z* 191) of extracted mycelia of various species (indicated in panel C) that were cultured in the presence of D_2-**10**; C) investigated species within and outside of the family *Clitocybaceae.* [a]Assignment of the species to this family follows He et al.[22]; [b]Phylogenetic clades within the Clitocybaceae family; [c]major product or not identified/trace quantity in methanolic extract. Synonymous species names are: *Collybia rivulosa* (syn. *Clitocye rivulosa*), *Collybia phyllophila* (syn. *Clitocybe phyllophila*), *Collybia odora* (syn. *Clitocybe odora*), *Collybia nuda* (syn. *Lepista nuda*), and *Clitocybe nebularis* (syn. *Lepista nebularis*). The psilocybin producer *Psilocybe cubensis* and the reishi mushroom *Ganoderma lucidum* both have a long tradition of being consumed by humans as recreational drug and as a medicinal mushroom of the eastern medicine, respectively.

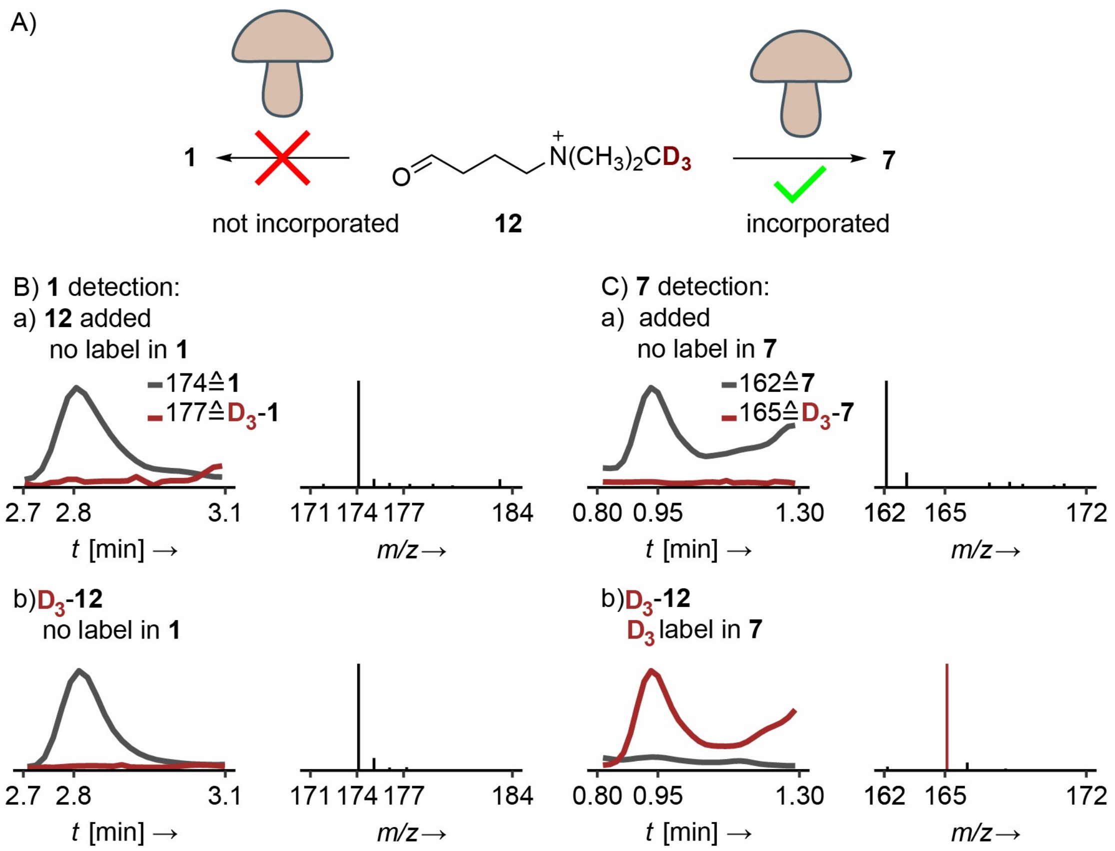

Figure S3. Biosynthetic divergence of the **1** and **7** pathways, shown by the incorporation of 4-(trimethylamino)butanal (**12**): Shown are EICs (left) and mass spectra (right) of extracted *C. rivulosa* mycelia, supplied with **12** (for control) or D3-**12**.

A) Interpretation of feeding experiment.

B) Incorporation of **12** or D3-**12**, respectively, into **1**;

C) incorporation of **12** or D3-**12** into **7**. The second peak that appears after approximately 1.2 min and that also shows stable isotope incorporation plausibly represents the methanol adduct of **12**/D3-**12** (*m*/*z* 130 or 133 + 32 = 162 or 165) that forms during extraction and shows the same *m*/*z* as **7**.

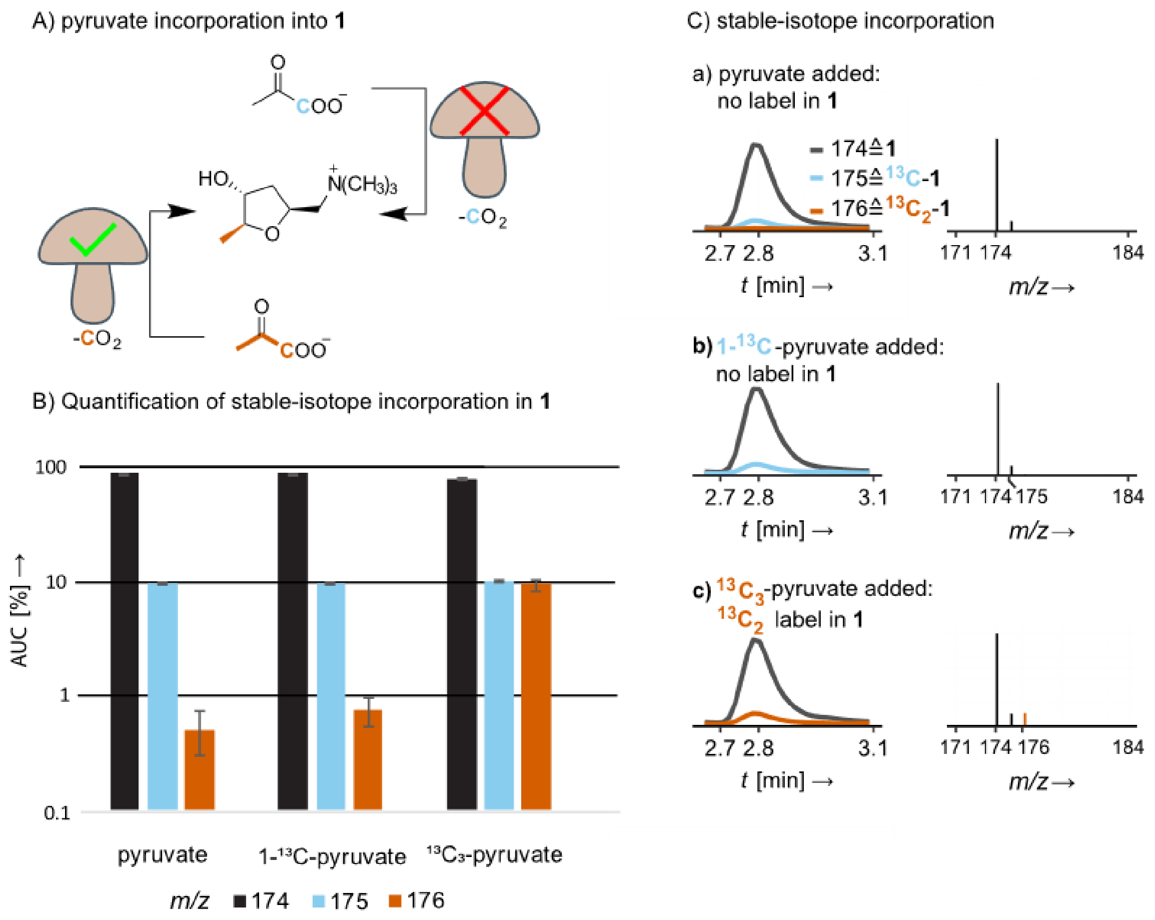

Figure S4. Full pyruvate stable-isotope labeling to identify the origin of carbon atoms C2 and 2-CH_3 in **1**.

A) depicts the found/disproven stable-isotope incorporation reaction of pyruvate into **1**.

B) Quantification of stable-isotope incorporation in **1**. Bar diagrams show the averaged percent of the areas under curve of the *m*/*z* 174, 175, and 176 peaks. Note the logarithmic y-axis. Labeled compounds or unlabeled controls were added at 5 mM final concentration to the cultures. Error bars indicate the standard deviation (n = 3).

C) EICs (left) and mass spectra (right) of extracted *C. rivulosa* mycelia cultured in the presence of unlabeled compounds for control or stable isotope-labeled compounds are shown.

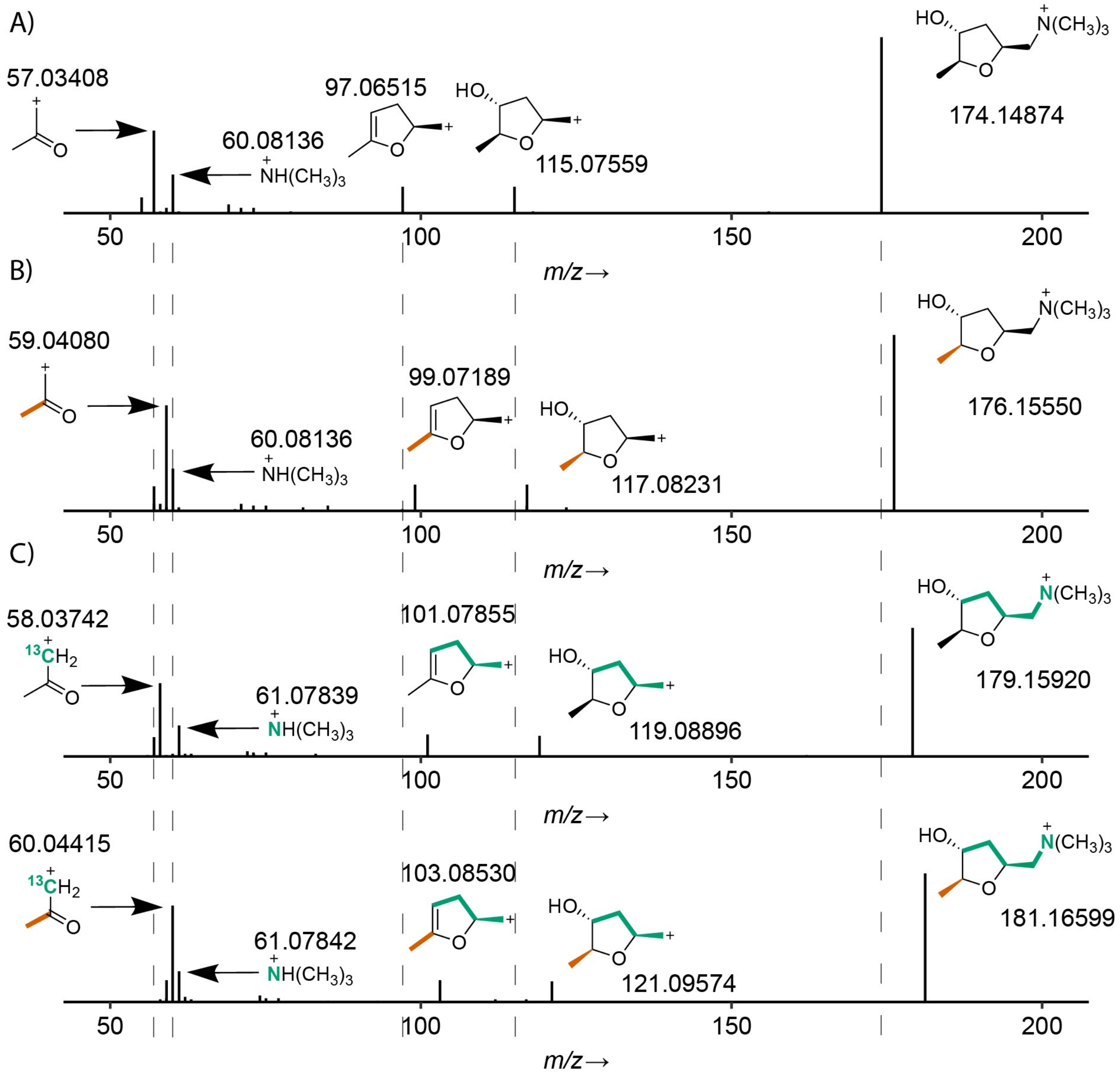

Figure S5. MS/MS spectra and assigned fragments to trace stable isotope incorporation into the muscarine (**1**) backbone. Green: L-lysine-derived isotopically labeled atoms; orange: L-alanine-derived isotopically labeled atoms.

A) unlabeled **1**,

B) $^{13}C_2$-**1** from 2,3-$^{13}C_2$-L-alanine,

C) $^{13}C_4{}^{15}N$ -**1** from U-$^{13}C^{15}N$-L-lysine,

D) $^{13}C_6{}^{15}N$-**1** from U-$^{13}C^{15}N$-L-lysine and 2,3-$^{13}C_2$-L-alanine incorporation. For corresponding *m/z* values, please see Figure S6.

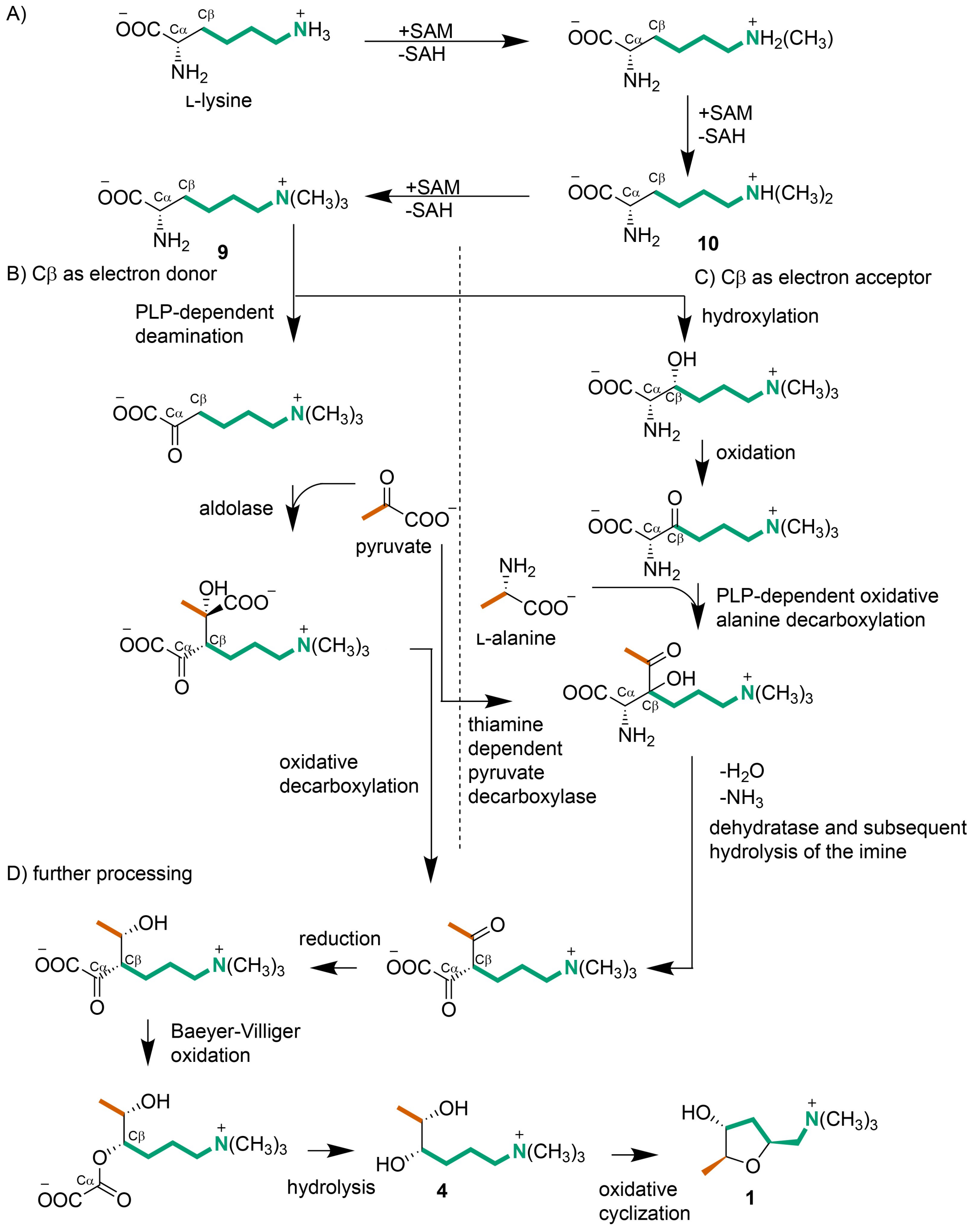

Figure S6. Proposed biosynthetic pathways for **1** and expected enzymatic activities.

A) Initiation of the biosynthesis through non-peptidyl L-lysine trimethylation to ε-*N*,*N*,*N*-trimethyl-L-lysine (**9**) according to this study,

B) pathway involving the incorporation of pyruvate,

C) pathway involving the incorporation of L-alanine,

D) post-assembly processing. Green: L-lysine-derived isotopically labeled atoms; orange: L-alanine-derived isotopically labeled atoms.

Background information

A) Based on stable isotope-labeled compounds, the biosynthesis of **1** was shown to begin with the iterative trimethylation of non-peptidyl-L-lysine to **9** catalyzed by a SAM-dependent methyltransferase. Trimethylation of L-lysine as peptidyl-L-lysine to peptidyl-**9** is common for histone modification processes, carried out by SET domain-containing methyltransferases (SET-MTs). However, SET-MTs are rarely involved in secondary metabolism. Only in four cases SET-MTs were shown or have been predicted to transform substrates in the biosynthesis of natural products.[41-44] In the case of malleilactone, BurB methylates free methionine, generating *S*-methylmethionine[41]; for flavunoidine biosynthesis, FlvH is predicted to dimethylate free L-lysine to **10**;[42] to accomplish epichloëcycline biosynthesis, GigC dimethylates peptidyl-L-lysine to peptidyl-**10.**[43] Finally, in goadvionin biosynthesis GdvMT, is proposed to trimethylate free L-lysine to **9**.[44] As the ε-trimethylation of L-lysine residues is commonly performed by SET-MTs, and these have been shown to act on non-peptidyl substrates and perform transformations in secondary metabolism, we hypothesize that a SET-MT may generate **9** to supply **1** biosynthesis.

B) The Cβ of L-lysine hypothetically serves as electron donor during coupling: in this case, the deamination of **9** generates a functional group capable of serving an electron donor at Cβ of L-lysine. Coupling could proceed with pyruvate as the electron acceptor, catalyzed by an aldolase[45] to establish the **1** backbone. Further processing includes the removal of the COOH group of pyruvates by oxidative decarboxylation, generating a ketone,[46] followed by further processing steps (see section D).

C) In a second scenario, Cβ of L-lysine may serve as electron acceptor during coupling: hydroxylation of **9** to *S*,*S*-**11** and subsequent oxidation of the hydroxyl group to the ketone would generate a functional group serving as an electron acceptor at Cβ of L-lysine. Coupling with L-alanine could proceed through a PLP-dependent oxidative decarboxylation of L-alanine, similar to the reaction catalyzed by CuaB during curvulamine biosynthesis.[47] Alternatively, the coupling could also proceed through a PLP-dependent L-alanine decarboxylase, generating an amine that would additionally require deamination to yield the same adduct.[48] Yet another alternative includes the use of pyruvate, instead of L-alanine, by a pyruvate decarboxylase generating the same product.[49]

D) Cleavage of the C-C-bond (derived from L-lysine's Cα-Cβ) could proceed via a Baeyer-Villiger monooxygenase.[50-52] Hydrolysis of the resulting ester would i) complete the removal of the excess C_2 fragment, ii) liberate oxalic acid and iii) generate **4**. This compound might become oxidatively cyclized. The step may be catalyzed either by a cytochrome P450 enzyme, as shown for aureothin and platensimycin biosynthesis,[53,54] or by α-ketoglutarate-dependent dioxygenases, as found in the biosynthesis of loline.[55]

.

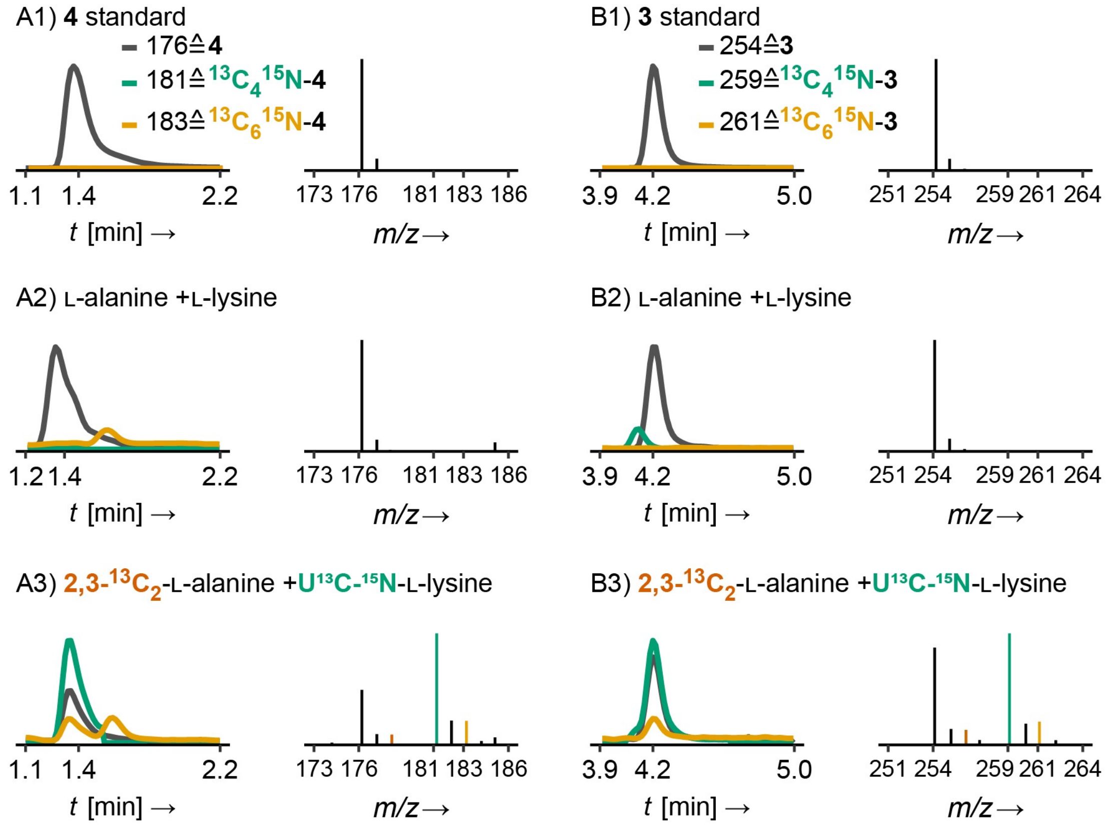

Figure S7. Mass spectrometric analysis of 4´-phosphomuscarine (**3**) and muscaridine (**4**). Extracted ion chromatograms (EIC) and mass spectra are shown. For analysis, methods I and II (Table S4), respectively, were applied. Green: EICs to detect L-lysine-derived isotopically labeled atoms; orange: EICs to detect L-alanine derived isotopically labeled atoms; amber: EICs to detect both L-lysine- and L-alanine-derived atoms after simultaneous feeding of both labeled compounds.

Table S1. Incorporation of the C_2 fragment into muscarine (1). For corresponding extracted ion chromatograms and bar diagrams, please see Figure 6.

m/z	**pyruvate**	**1-^{13}C pyruvate**	**$^{13}C_3$-pyruvate**	**acetate**	**1,2-$^{13}C_2$-acetate**	**L-alanine**	**2,3-^{13}C- L-alanine**	**$^{13}C_3$-pyruvate + L-lysine**
174	89.75 ± 0.09	89.74 ± 0.37	80.38 ± 1.03	90.06 ± 0.98	88.23 ± 0.68	89.33 ± 0.32	75.40 ± 0.38	76.85 ± 0.95
175	9.74 ± 0.23	9.51 ± 0.21	10.17 ± 0.20	9.36 ± 1.04	10.72 ± 0.62	10.03 ± 0.23	10.68 ± 0.63	11.10 ± 0.60
176	0.51 ± 0.21	0.75 ± 0.21	9.45 ± 1.07	0.57 ± 0.06	1.05 ± 0.06	0.64 ± 0.10	13.92 ± 0.71	12.04 ± 0.87

Table S2. Incorporation of stable isotopes into muscarine (1), traced by MS/MS. Green: L-lysine derived stable isotopes; orange: L-alanine derived stable isotopes For corresponding spectra, please refer to Figure S5. Green: L-lysine-derived isotopically labeled atoms; orange: L-alanine derived isotopically labeled atoms

	Fragment structure	Calculated *m/z*	Found *m/z*	Sumformula	δ ppm
	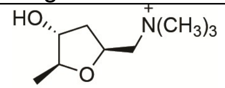	174.14886	174.14874	$C_9H_{20}O_2N$	-0.663
	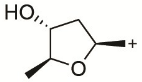	115.07536	115.07559	$C_6H_{11}O_2$	2.032
	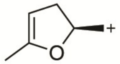	97.06479	97.06515	C_6H_9O	3.694
	$\overset{+}{N}H(CH_3)_3$	60.08078	60.08136	$C_3H_{10}N$	9.722
**1**	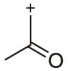	57.03349	57.03408	C_3H_5O	10.321
	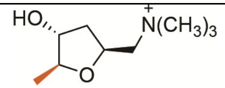	176.15556	176.15550	$C_7{}^{13}C_2H_{20}O_2N$	-0.369
	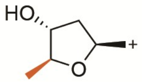	117.08207	117.08231	$C_4{}^{13}C_2H_{11}O_2$	2.086
	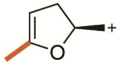	99.07150	99.07189	$C_3{}^{13}C_2H_8N^{15}N$	-0.548
	$\overset{+}{N}H(CH_3)_3$	60.08078	60.08136	$C_3H_{10}N$	9.722
$^{13}C_2$-**1**	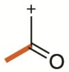	59.04020	59.04080	$C^{13}C_2H_5O$	10.146

	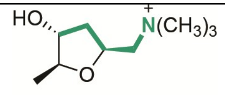	179.15931	179.15920	$C_5{}^{13}C_4H_{20}O_2{}^{15}N$	-0.611
	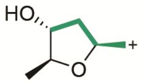	119.08878	119.08896	$C_2{}^{13}C_4H_{11}O_2$	1.551
	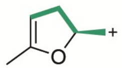	101.07821	101.07855	$C^{13}C_4H_8N^{15}N$	-1.028
	$\overset{+}{N}H(CH_3)_3$	61.07781	61.07839	$C_3H_{10}{}^{15}N$	9.483
$^{13}C_4{}^{15}N$-**1**	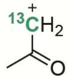	58.03685	58.03742	$C_2{}^{13}CH_5O$	9.888
	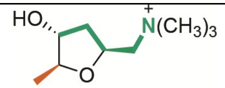	181.16602	181.16599	$C_3{}^{13}C_6H_{20}O_2{}^{15}N$	-0.161
	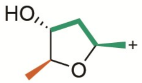	121.09549	121.09574	$^{13}C_6H_{11}O_2$	2.106
	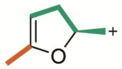	103.08492	103.08530	$^{13}C_6H_9O$	3.684
	$\overset{+}{N}H(CH_3)_3$	61.07781	61.07842	$C_3H_{10}{}^{15}N$	9.974
$^{13}C_6{}^{15}N$-**1**	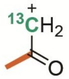	60.04356	60.04415	$^{13}C_3H_5O$	9.897

Table S3. Fungal species used during this study. Strains were obtained from the Jena Microbial Resource Collection (JMRC).

**Species**	**JMRC number**
Collybia rivulosa (=*Clitocybe rivulosa)*	FSU 9986
Collybia phyllophila<br><br>(=*Clitocybe phyllophila*, *Clitocybe cerussata)*	SF015747
Collybia odora (=*Clitocybe odora)*	SF010328
Collybia nuda (=*Lepista nuda)*	ST020799
Clitocybe nebularis (=*Lepista nebularis)*	STH00411
Ganoderma lucidum	SF012602
Psilocybe cubensis	FSU 12407

Table S4. Chromatographic methods and instruments. Analytical (anal.), semi-preparative (semi-prep.), preparative (prep.) liquid chromatography and protein chromatography (prot. chrom.). Acronyms: ACN: acetonitrile, FA: formic acid, MBP: maltose brinding protein, MeOH: methanol.

Method	Column	Solvents	Flow [ml min^{-1}]	Column [°C]	Gradient	Instrument[a]
I (anal.)	Supelco Ascentis Express 90 Å F5 (2.1 × 100 mm, 2 µm), with guard (2.1 × 5 mm, 2 µm)	A: H_2O (0.1% FA)<br>B: ACN	0.4	35	0-1 min B: 1 %<br>1-4 min B: 1-95 %<br>4-4.5 min B: 95 %	1, 2
II (anal.)	Zorbax HILIC Plus (4.6 × 100 mm, 3.5 µm)	A: H_2O (0.1% FA)<br>B: ACN	1	35	0-0.5 min B:80 %<br>0.5-5 min B: 80-10 %<br>5.5-6 min B: 80 %	1
III (prep.)	Phenomenex Luna PFP(2) (21.2 × 250 mm; 5 µm; 100 Å pore size), with guard (21.2 × 15 mm; 5 µm)	A: H_2O (0.1% FA)<br>B: MeOH	25	RT	0-10 min: 1% B	3
IV (prep.)	Phenomenex Luna PFP(2) (21.2 × 250 mm; 5 µm; 100 Å pore size), with guard (21.2 × 15 mm; 5 µm)	A: H_2O (0.1% FA)<br>B: ACN	15	RT	0-3 min: 5<br>3-10 min: 5-50<br>10-17 min: 50	3
V (prep.)	Phenomenex Eclipse XDB-C8 (21.2 × 250 mm; 7 µm), with guard (21.2 × 15 mm; 7 µm)	A: H_2O (0.1% FA)<br>B: ACN	20	RT	0-20 min: 5-20 % B	3
VI (semi-prep.)	Zorbax HILIC Plus (4.6 × 100 mm, 3.5 µm)	A: H_2O (0.1% FA)<br>B: ACN	3	23	0-2.5 min: 80-10 % B<br>2.5-3.5 min: 10 % B	4

VII (prot. chrom.)	two serially connected Cytiva MBP-Trap HP 1 mL columns (bed volume)	A: 20 mM Tris-HCl; pH 8; 200 mM NaCl<br>B: 20 mM Tris-HCl; pH 8; 200 mM NaCl, 5 mM maltose	Sample applic.: 0.5<br>wash/elution: 2	RT	Sample application:<br>0-30 min: 0% B<br><br>Wash/Elution:<br>30-36.5 min: 0% B<br>36.5-41.5 min: 100% B	5

[a] See section „Liquid Chromatography" in the Experimental Section for details on the instruments.

References

[18] K. Nitta, R. J. Stadelmann, C. H. Eugster, *Helv. Chim. Acta* **1977**, *60*, 1747–1753.

[19] S. Obermaier, M. Müller, *Angew. Chem. Int. Ed.* **2020**, *59*, 12432–12435.

[20] S. Obermaier, PhD thesis, Albert-Ludwigs-Universität, Freiburg (Germany), **2019**.

[22] Z.-M. He, Z.-H. Chen, T. Bau, G.-S. Wang, Z. L. Yang, *Fungal Div.* **2023**, *123*, 1–47.

[24] A. Kazaks, M. Makrecka-Kuka, J. Kuka, T. Voronkova, I. Akopjana, S. Grinberga, O. Pugovics, K. Tars, *Prot. Expr. Purif.* **2014**, *104*, 1–6.

[25] A. H. K. Al Temimi, B. J. G. E. Pieters, Y. V. Reddy, P. B. White, J. Mecinović, *Chem. Commun.* **2016**, *52,* 12849–12852.

[30] M. Moser, Die Pilze Mitteleuropas, 4th ed., Klinkhardt, Bad Heilbrunn, **1960**, pp. 58–62.

[31] N. M. T. Lourenço, C. M. Monteiro, C. A. M. Afonso, *Eur. J. Org. Chem.* **2010**, *2010*, 6938–6943.

[32] M. A. Gamal-Eldina, D. H. Macartney, *Org. Biomol. Chem.* **2013**, *11*, 488–495.

[33] D. Zelencova-Gopejenko, A. Grandane, E. Loza, D. Lola, A. Sipola, E. Liepinsh, P. Arsenyan, K. Jaudzems, *ACS Med. Chem. Lett.* **2022** 13, 1723–1729.

[34] J. W. Lamppa, S. A Tanyos, K. E. Griswold, *J. Biotechnol.* **2013**, *164*, 1–8.

[35] Y. V. Reddy, A. H. K. Al Temimi, P. B. White, J. Mecinović, *Org. Lett.* **2017**,*19*, 400–403.

[36] M. Hassan, S Morimoto, H. Murakami, T. Ichiyanagi, N. Mori, *Biosci. Biotechnol. Biochem.* **2007**, *71*, 1439–1446.

[37] G. R. Fulmer, A. J. Miller, N. H. Sherden, H. E. Gottlieb, A. Nudelman, B. M. Stoltz, J. E. Bercaw, K. I. Goldberg, *Organometallics* **2010**, *29*, 2176–2179.

[38] KEGG pathway (lysine) accessible under: https://www.genome.jp/kegg-bin/show_pathway?abp00300.

[39] KEGG pathway (carnitine) accessible under: https://www.genome.jp/kegg-bin/show_pathway?abp00310.

[40] T. Laubach, A. von Haeseler, M. J. Lercher, *BMC Bioinformatics* **2012**, *13*:110.

[41] F. Trottmann, K. Ishida, J. Franke, A. Stanišić, M. Ishida-Ito, H. Kries, G. Pohnert, C. Hertweck, *Angew. Chem. Int. Ed.* **2020**, *59*, 13511–13515.

[42] D. A. Yee, T. B. Kakule, W. Cheng, M. Chen, C. T. Y. Chong, Y. Hai, L. F. Hang, Y.-S. Hung, N. Liu, M. Ohashi, I. C. Okorafor, Y. Song, M. Tang, Z. Zhang, Y. Tang, *J. Am. Chem. Soc.* **2020**, *142*, 710–714.

[43] W. Zhang, N. T. Forester, P. Chettri, M. Heilijgers, W. J. Mace, E. Maes, Y. Morozova, E. R. Applegate, R. D. Johnson, L. J. Johnson, *J. Agric. Food Chem.* **2023**, *71*, 13965–13978.

[44] R. Kozakai, T. Ono, S. Hoshino, H. Takahashi, Y. Katsuyama, Y. Sugai, T. Ozaki, K. Teramoto, K. Teramoto, K. Tanaka, I. Abe, S. Asamizu, H. Onaka, *Nat Chem* **2020**, *12*, 869–877.

[45] H. Zhao, *RSC Adv.* **2024**,*14*, 25932–25974.

[46] B**.** Fu, E. P. Balskus, *Curr. Opin. Biotechnol.* **2020**, *65*, 94–101.

[47] G. Z. Dai, W. B. Han, Y. N. Mei, K. Xu, R. H. Jiao, H. M. Ge, R. X. Tan, *Proc. Natl. Acad. Sci. U.S.A.* **2019**, *117*, 1174–1180.

[48] Y.-L. Du, K. S. Ryan, *Nat. Prod. Rep.* **2019**, 36, 430–457.

[49] M. Brovetto, D. Gamenara, P. S. Méndez, G. A. Seoane, *Chem. Rev.* **2011**, *111*, 4346–4403.

[50] G. de Gonzalo, M. D. Mihovilovic, M. W. Fraaije, *ChemBioChem* **2010**, *11*, 2208–2231.

[51] H. Leisch, K. Morley, Peter C. K. Lau, *Chem. Rev.* **2011**, *111*, 4165–4222.

[52] D. E. Torres Pazmiño, H. M. Dudek, M. W. Fraaije, *Curr. Opin. Chem. Biol.* **2010**, *14*, 138–144.

[53] M. Richter, N. Traitcheva, U. Knüpfer, C. Hertweck, *Angew. Chem. Int. Ed.* **2008**, *47*, 8872–8875.

[54] J. D. Rudolf, L. Dong, X. Zhang, H. Renata, B. Shen, *J. Am. Chem. Soc.* **2018**, *140*, 12349–12353.

[55] J. Pan, M. Bhardwaj, B. Zhang, W. Chang, C. L. Schardl, C. Krebs, R. B. Grossman, J. M. Bollinger, Jr., *Biochemistry*, **2018**, *57*, 2074–2083.
